# Drug‐Induced Cuproptosis Defines the Therapeutic Window of Celecoxib in Intervertebral Disc Degeneration via the HSP90‐RBX1 Axis

**DOI:** 10.1002/advs.75527

**Published:** 2026-05-04

**Authors:** Youfeng Guo, Hongju Xiao, Shenghao Ba, Yu Zhou, Bijun Wang, Bin Yu, Yufeng Huang, Haihong Zhao, Zhefan Stephen Chen, Na Shen, Zhaoyu Ba, Desheng Wu

**Affiliations:** ^1^ Department of Spine Surgery School of Medicine Shanghai East Hospital Tongji University Shanghai China; ^2^ Anhui Medical University Hefei Anhui China; ^3^ School of Life Sciences The Chinese University of Hong Kong Shatin, N.T. Hong Kong SAR China; ^4^ Department of Orthopedics School of Medicine Yangpu Hospital Tongji University Shanghai China; ^5^ Gerald Choa Neuroscience Institute The Chinese University of Hong Kong Shatin, N.T. Hong Kong SAR China; ^6^ Department of Hematology Jiangsu Province Hospital The First Affiliated Hospital of Nanjing Medical University Nanjing Jiangsu China

**Keywords:** celecoxib, cuproptosis, HSP90, intervertebral disc degeneration, RBX1

## Abstract

Intervertebral disc degeneration (IDD) is a major cause of low back pain, yet the biological effects of commonly used non‐steroidal anti‐inflammatory drugs (NSAIDs) on disc cells remain poorly understood. Celecoxib is widely prescribed for IDD‐related pain, but its direct influence on IDD has not been systematically examined. Here, we identify a concentration‐dependent biphasic effect of celecoxib on nucleus pulposus (NP) cells and uncover the mechanism that converts celecoxib from protective to detrimental. Using interleukin‐1β‐stimulated NP cells and rat IDD models, we show that low‐dose celecoxib (≤20 µm) suppresses inflammation and preserves extracellular matrix (ECM). In contrast, high‐dose celecoxib (>20 µm) activates a previously unrecognized heat shock protein 90 (HSP90)/RING‐box protein 1 (RBX1)/cuproptosis axis, leading to copper accumulation, mitochondrial stress, and ECM degradation. Mechanistically, elevated celecoxib induces HSP90 upregulation, which stabilizes RBX1 by reducing its K48‐linked ubiquitination. Accumulated RBX1 promotes ATPase copper transporting beta (ATP7B) and its regulator copper metabolism domain containing 1 (COMMD1) degradation, thereby triggering cuproptosis. Pharmacologic inhibition of HSP90 or cuproptosis effectively reverses the detrimental effects of high‐dose celecoxib in vivo. Together, these findings define a strict therapeutic window for celecoxib in IDD and reveal a novel HSP90/RBX1‐mediated cuproptosis pathway that mediates its dual effects.

## Graphical abstract

This study reveals the dual role of celecoxib in intervertebral disc degeneration. While low concentrations are protective, high concentrations induce toxicity by upregulating HSP90, which synergizes with USP15 to deubiquitinate and stabilize RBX1. This leads to degradation of COMMD1/ATP7B, copper dyshomeostasis, and ultimately, cuproptosis.

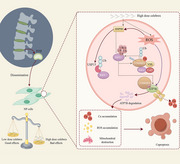

## 1 Background

Chronic low back pain represents a substantial global health burden, with intervertebral disc degeneration (IDD) identified as its principal pathological cause [1, 2]. The pathogenesis of IDD is a dynamic and intricate process characterized by inflammation, dysregulation of the extracellular matrix (ECM), and cellular apoptosis [3, 4, 5, 6]. The overproduction of pro‐inflammatory mediators, such as interleukin‐1β (IL‐1β) and tumor necrosis factor α (TNF‐α), perpetuates a detrimental cycle by directly sensitizing neural pathways and disrupting matrix homeostasis within nucleus pulposus (NP) cells [7, 8]. Thus, understanding IDD mechanisms to disrupt this cycle is clinically vital.

Clinically, the selective cyclooxygenase‐2 (COX‐2) inhibitor celecoxib is a first‐line treatment for IDD‐related pain due to its potent anti‐inflammatory and analgesic effects [9, 10, 11, 12, 13, 14]. Its primary mechanism involves inhibiting COX‐2 to reduce prostaglandin production [15]. Nevertheless, a significant paradox persists: despite its widespread utilization, the specific effects of celecoxib on disc tissue, particularly NP cells, remain inadequately understood, with literature reports occasionally presenting conflicting findings. This suggests its actions may be more complex than simple COX‐2 inhibition. Indeed, non‐steroidal anti‐inflammatory drugs (NSAIDs) are known to have concentration‐dependent dual effects [16, 17]. At elevated concentrations, these substances may inadvertently activate unanticipated and potentially deleterious signaling pathways through “off‐target” effects; however, the precise mechanisms underlying these phenomena within the context of IDD remain insufficiently understood.

Meanwhile, cuproptosis, a newly defined copper‐dependent cell death, is emerging as a key player in degenerative diseases [18, 19, 20]. In contrast to apoptosis, this process entails the binding of copper to lipoylated proteins within the tricarboxylic acid (TCA) cycle, leading to oligomerization and subsequent proteotoxic stress [21]. While linked to chemotherapy resistance in oncology [22, 23, 24], it is also implicated in IDD. Its regulation involves factors like Copper Metabolism Domain Containing 1 (COMMD1), which modulates copper homeostasis via the cullin‐RING E3 Ub ligase (CRL) complexes [25]. RING‐box protein 1 (RBX1), a core CRL component, is crucial for ubiquitination [26, 27, 28], raising the question: could RBX1 directly regulate cuproptosis?.

The potential for NSAIDs, particularly celecoxib, to directly initiate cell death mechanisms like cuproptosis, and the specific concentration conditions under which this may occur, remains completely uncharted. In this study, we elucidate a molecular switch driven by heat shock protein 90 (HSP90) that transforms celecoxib from a therapeutic agent to a deleterious one by activating RBX1‐mediated cuproptosis. Collectively, this study reveals three previously unrecognized mechanisms: (1) Celecoxib at supratherapeutic levels can induce cuproptosis in NP cells; (2) HSP90 stabilizes the RBX1 protein by inhibiting its degradation; and (3) RBX1 disrupts copper homeostasis by degrading COMMD1 and ATPase Copper Transporting Beta (ATP7B), with COMMD1 crucial for ATP7B stability. These findings contribute to a deeper understanding of the potential toxicity of celecoxib in IDD and offer a theoretical foundation for targeting the “HSP90/RBX1/cuproptosis” axis in clinical strategies.

## 2 Results

### 2.1 Celecoxib Exhibits Concentration‐Dependent Therapeutic Effects in IL‐1β‐Induced NP Cells and Rat IDD Models

**FIGURE 1:**
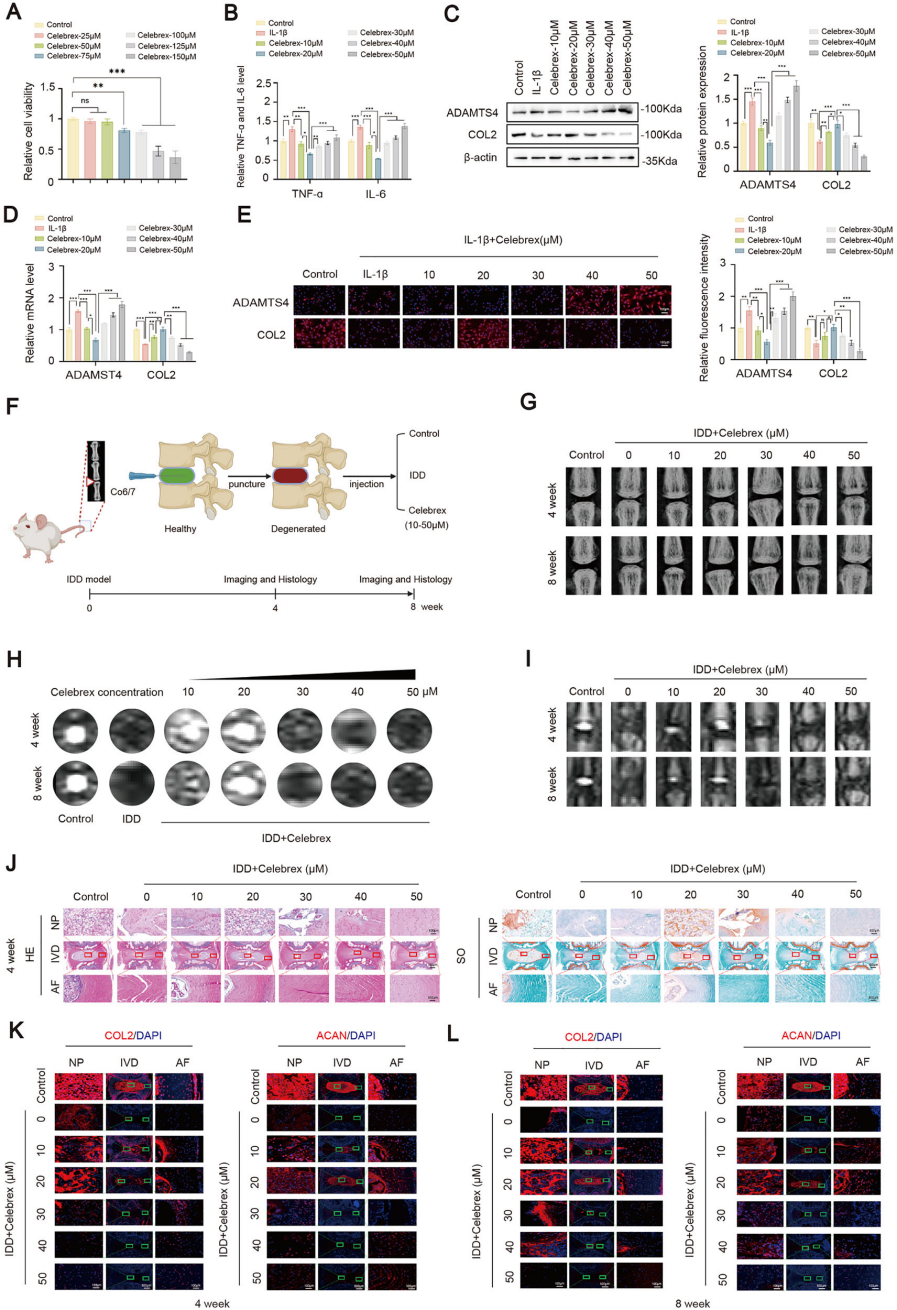
Effects of different concentration gradients of celecoxib on IDD. (A) Under IL‐1β (10 ng/mL) exposure, the effect of celecoxib pretreatment at different concentrations on cell viability for 48 h (*n* = 3). (B) Under IL‐1β (10 ng/mL) exposure, protein levels of inflammatory factors (TNF‐α and IL‐6) were detected by ELISA after treatment with different concentrations of celecoxib for 48 h (*n* = 3). (C) Under IL‐1β (10 ng/mL) exposure, protein levels of ADAMTS4 and COL2 were detected after treatment with different concentrations of celecoxib for 48 h, with semi‐quantitative analysis of band grayscale values using ImageJ (*n* = 3). (D) Under IL‐1β (10 ng/mL) exposure, mRNA levels of ADAMTS4 and COL2 were detected after treatment with different concentrations of celecoxib for 48 h (*n* = 3). (E) Under IL‐1β (10 ng/mL) exposure, levels of corresponding degeneration markers (ADAMTS4 and COL2) were analyzed by IF staining after treatment with different concentrations of celecoxib for 48 h, with semi‐quantitative analysis using ImageJ (*n* = 3). (F) Schematic diagram of the animal experiment. (G) Representative X‐ray scans and DHI index of IVDs at 4‐ and 8‐weeks post‐intervention (*n* = 6). (H, I) Representative MRI scans and their MRI index calculations obtained at 4‐ and 8‐weeks post‐intervention (*n* = 6). (J) Representative HE staining and SO staining images at 4 weeks post‐intervention (*n* = 6). (K, L) COL2 and ACAN IF staining detection and quantitative analysis of IF staining at 4‐ and 8‐weeks post‐intervention (*n* = 6). All data are presented as mean ± SD. Comparisons between two groups were performed using an unpaired two‐tailed Student's *t*‐test or ANOVA followed by Tukey's post hoc test. A *p*‐value less than 0.05 was considered statistically significant. ^*^ indicates *p* < 0.05, ^**^ indicates *p* < 0.01, ^***^ indicates *p* < 0.001, while “ns” indicates a lack of statistical significance. IDD, Intervertebral Disc Degeneration; IL‐1β, Interleukin‐1 beta; TNF‐α, Tumor Necrosis Factor‐alpha; IL‐6, Interleukin‐6; ELISA, Enzyme‐Linked Immunosorbent Assay; Wb, Western blot; ADAMTS4, A Disintegrin and Metalloproteinase with Thrombospondin Motifs 4; COL2, Type II Collagen; qPCR, quantitative Polymerase Chain Reaction; IF, Immunofluorescence; ECM, Extracellular Matrix; DHI, Disc Height Index; IVD, Intervertebral Disc; MRI, Magnetic Resonance Imaging; HE, Hematoxylin and Eosin; SO, Safranin O; NP, Nucleus Pulposus; ACAN, Aggrecan; SD, Standard Deviation; ANOVA, one‐way analysis of variance.

To ascertain the optimal therapeutic concentration of celecoxib for IDD, we initially evaluated its effects on the viability of NP cells. The results of the CCK‐8 assays demonstrated that celecoxib did not exhibit significant cytotoxicity at concentrations up to 50 µm. However, concentrations exceeding this threshold resulted in a pronounced decrease in cell viability, as illustrated in Figure 1A. Therefore, the 10–50 µm range was selected for subsequent experiments investigating its protection against IL‐1β (10 ng/mL)‐induced inflammation and degeneration. The results demonstrated a clear concentration‐dependent therapeutic effect. Specifically, ELISA showed that 10 and 20 µm celecoxib significantly suppressed the secretion of the pro‐inflammatory factors TNF‐α and IL‐6 compared to the IL‐1β model group (Figure 1B). Simultaneously, analyses using Western blot (Wb), quantitative polymerase chain reaction (qPCR), and immunofluorescence (IF) consistently demonstrated that these concentrations effectively counteracted IL‐1β‐induced catabolic alterations. This was evidenced by a significant reduction in the expression of A Disintegrin and Metalloproteinase with Thrombospondin Motifs 4 (ADAMTS4) and an enhancement in the synthesis of the anabolic key component, collagen type II (COL2) (Figure 1C–E). Notably, this beneficial effect peaked at 20 µm, providing the strongest anti‐inflammatory and ECM‐protective outcomes. However, further increasing the concentration to 30, 40, and 50 µm led to a gradual weakening of all therapeutic benefits, including diminished inhibition of inflammatory factors, a rebound in ADAMTS4 expression, and a significant decline in the promotion of COL2 synthesis (Figure 1C–E). These findings indicate that celecoxib exerts a biphasic effect on degenerated NP cells, with an optimal concentration window of 10–20 µm; lower concentrations have limited efficacy, while concentrations exceeding 20 µm yield diminishing returns.

To verify the therapeutic effect of celecoxib in vivo, we established a rat tail needle puncture degeneration model and administered intervention via intradiscal injection of different concentrations (10–50 µm) of celecoxib (Figure 1F). A comprehensive evaluation was conducted at 4 and 8 weeks post‐treatment utilizing imaging, histomorphological, and molecular biological methodologies. The imaging analysis (Figure 1G–I; Figure S1A,B) demonstrated that celecoxib mitigated the loss of disc height index (DHI) and reduction in water content in a concentration‐dependent manner. Notably, the 20 µm treatment group most effectively preserved the DHI and magnetic resonance imaging (MRI) index, exhibiting values significantly superior to those of the IDD group and closely approximating control levels. The 10 µm group displayed a moderate effect, while the high‐concentration treatment groups (30–50 µm) resembled the IDD group, showing a markedly diminished protective effect. Histological analysis (Hematoxylin and Eosin (HE) and Safranin O/Fast Green (SO) staining, Figure 1J; Figure S1C–E) further confirmed that 20 µm celecoxib effectively preserved the gel‐like characteristics and proteoglycan content of the NP and improved the regularity of the annulus fibrosus (AF) structure. The 10 µm group also showed significant improvement, albeit weaker than the 20 µm group. In contrast, the high‐concentration groups (30–50 µm) showed no significant difference in histological scores compared to the IDD group, with degenerative features remaining apparent. At the molecular mechanism level, tissue IF (Figure 1K–L; Figure S1F) and Wb (Figure S1G) results were consistent, indicating that 20 µm celecoxib treatment most effectively enhanced the expression of COL2 and aggrecan (ACAN) in NP tissue while significantly downregulating the level of the degeneration‐related protease ADAMTS4. The 10 µm concentration was also effective but weaker than 20 µm. When the concentration exceeded 20 µm, its promotive effect on ECM synthesis and inhibitory effect on catabolism were sharply attenuated.

### 2.2 Mechanism of Weakened Therapeutic Effect at High Celecoxib Concentrations: Activation of Cuproptosis Pathway Based on Quantitative Proteomics Analysis

**FIGURE 2:**
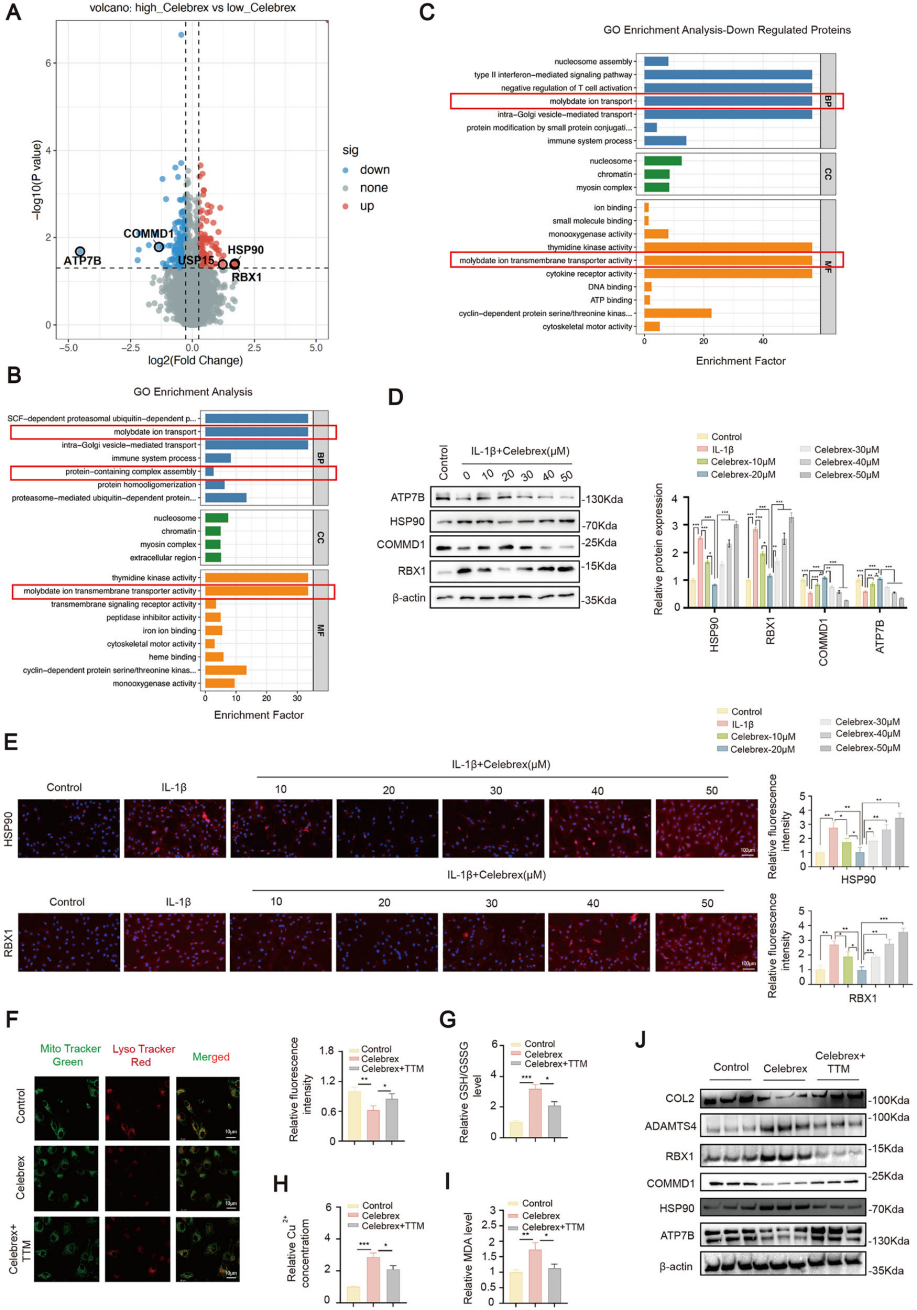
Investigation of the mechanism underlying the attenuated therapeutic effect of high‐concentration celecoxib.​​ (A) Volcano plot of differentially expressed proteins identified by quantitative proteomic analysis. (B, C) Quantitative proteomic comparison between NP cells treated with high‐concentration (50 µm) and low‐concentration (20 µm) celecoxib, followed by GO analysis of differentially expressed proteins. (D) Protein levels of ATP7B, HSP90, COMMD1, and RBX1 detected by Wb after treatment with different concentrations of celecoxib for 48 h in the presence of IL‐1β (10 ng/mL), with semi‐quantitative analysis of band grayscale values using ImageJ (*n* = 3). (E) Protein levels of HSP90 and RBX1 analyzed by IF staining after treatment with different concentrations of celecoxib for 48 h in the presence of IL‐1β (10 ng/mL), with semi‐quantitative analysis using ImageJ (*n* = 3). (F–I) Levels of cuproptosis in NP cells treated as indicated were determined by assessing mitophagy (F), GSH/GSSG ratio (G), Cu^2^^+^ level (H), and MDA content (I) (*n* = 3). (J) Protein levels of ATP7B, HSP90, COMMD1, RBX1, COL2, and ADAMTS4 detected by Wb after treatment with or without TTM for 48 h in the presence of celecoxib (50 µm), with semi‐quantitative analysis of band grayscale values using ImageJ (*n* = 3). All data are presented as mean ± SD. Comparisons between two groups were performed using an unpaired two‐tailed Student's *t*‐test or ANOVA followed by Tukey's post hoc test. A *p*‐value less than 0.05 was considered statistically significant. ^*^ indicates *p* < 0.05, ^**^ indicates *p* < 0.01, ^***^ indicates *p* < 0.001, while “ns” indicates a lack of statistical significance. NP, Nucleus Pulposus; GO, Gene Ontology; Wb, Western blot; ATP7B, ATPase Copper Transporting Beta; HSP90, Heat Shock Protein 90; COMMD1, Copper Metabolism MURR1 Domain‐containing protein 1; RBX1, RING Box Protein 1; IF, Immunofluorescence; GSH, Reduced Glutathione; GSSG, Oxidized Glutathione; Cu^2^^+^, Copper Ion; MDA, Malondialdehyde; TTM, Tetrathiomolybdate; COL2, Type II Collagen; ADAMTS4, A Disintegrin and Metalloproteinase with Thrombospondin Motifs 4; SD, Standard Deviation; ANOVA, one‐way analysis of variance.

To investigate the mechanism underlying the diminished efficacy of celecoxib at concentrations above 20 µm, we conducted quantitative proteomic analysis on NP cells, comparing the protein profiles between the 20 and 50 µm treatment groups (Figure 2A–C; Figure S2A). The analysis revealed that high‐concentration celecoxib specifically activated the cuproptosis pathway, inducing significant reprogramming of the expression profile. Among the differentially expressed proteins, HSP90, the E3 ubiquitin ligase RBX1, and the Ubiquitin Specific Peptidase 15 (USP15) were significantly upregulated, while the copper homeostasis regulators COMMD1 and ATP7B were downregulated in the 50 µm group. Subsequent Gene Ontology (GO) and Kyoto Encyclopedia of Genes and Genomes (KEGG) enrichment analyses indicated that the differentially expressed proteins were significantly associated with pathways related to “molybdate ion transport” and “proteasome degradation” (Figure 2B,C; Figure S2A), suggesting a link to copper ion homeostasis. This implies that high‐dose celecoxib may trigger cuproptosis by upregulating HSP90/RBX1 and downregulating COMMD1/ATP7B, thereby counteracting its therapeutic effects. The expression changes of these key molecules and the cuproptosis phenotype were concentration‐dependent. Verification experiments across a detailed concentration gradient (10–50 µm) via Wb, qPCR, and IF (Figure 2D,E; Figure S2B,C) consistently showed that at therapeutic concentrations (10 and 20 µm), increasing celecoxib levels gradually decreased HSP90 and RBX1 expression while increasing COMMD1 and ATP7B. Beyond a concentration of 20 µm, this trend was reversed, with an increase in the expression of HSP90 and RBX1, and a decrease in COMMD1/ATP7B levels. Subsequently, key phenotypic markers related to cuproptosis were evaluated. Treatment with 50 µm celecoxib resulted in a significant inhibition of intracellular mitophagy (Figure 2F). Moreover, the group treated with high‐concentration celecoxib demonstrated an increased intracellular ratio of glutathione (GSH) to glutathione disulfide (GSSG), elevated levels of Cu^2^^+^, and a higher content of the lipid peroxidation product malondialdehyde (MDA) compared to the control group (Figure 2G–I). Notably, these phenotypic changes induced by high‐concentration celecoxib were effectively reversed by the cuproptosis‐specific inhibitor Tetrathiomolybdate (TTM), but exacerbated by the cuproptosis agonist Elesclomol (Figure 2J; Figure S2D,E).

### 2.3 Mechanistic Dissection of the HSP90‐RBX1‐COMMD1/ATP7B‐Cuproptosis Axis

**FIGURE 3:**
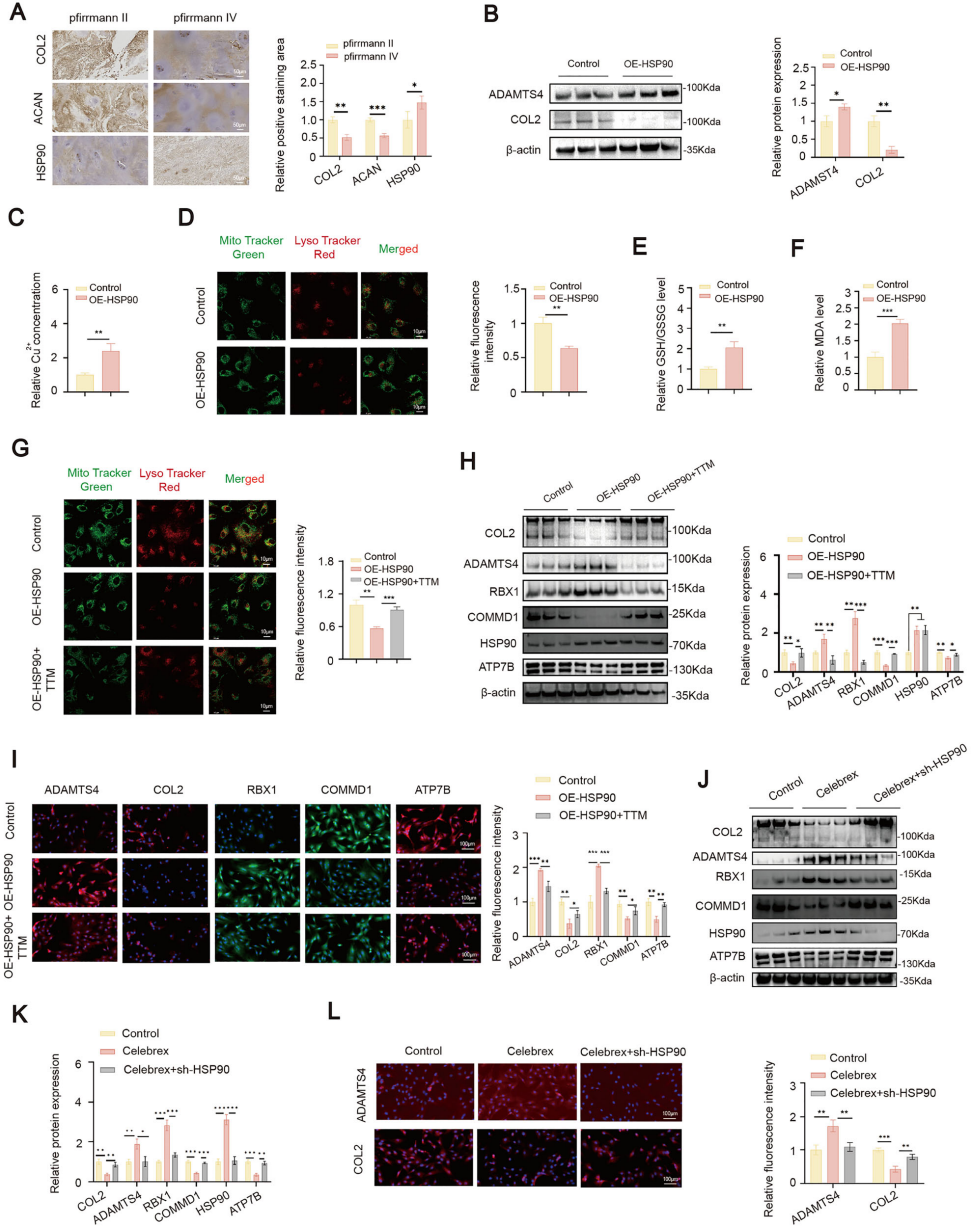
HSP90 drives NP cell degeneration via cuproptosis. (A) Representative IHC staining images and semi‐quantitative analysis of ECM (COL2 and ACAN) and HSP90 expression in human NP tissues section with different degeneration degrees (*n* = 3). (B) In vitro analysis of related (ADAMTS4 and COL2) levels by Wb in NP cells transfected as indicated. (C–G) The cuproptosis levels in NP cells transfected as indicated were determined by Cu^2+^ (C), mitophagy (D and G), GSH/GSSG (E) and MDA (F) levels. H‐L: In vitro analysis of related (ADAMTS4 and COL2), cuproptosis (ATP7B and COMMD1), HSP90 and RBX1 levels were determined by cellular IF staining (I and L) and Wb (H, J, K) of SD rat‐derived NP cells. All data are presented as mean ± SD. Comparisons between two groups were performed using an unpaired two‐tailed Student's *t*‐test or ANOVA followed by Tukey's post hoc test. A *p*‐value less than 0.05 was considered statistically significant. ^*^ indicates *p* < 0.05, ^**^ indicates *p* < 0.01, ^***^ indicates *p* < 0.001, while “ns” indicates a lack of statistical significance. HSP90, Heat Shock Protein 90; NP, Nucleus Pulposus; IHC, Immunohistochemistry; ECM, Extracellular Matrix; COL2, Type II Collagen; ACAN, Aggrecan; Wb, Western blot; ADAMTS4, A Disintegrin and Metalloproteinase with Thrombospondin Motifs 4; qPCR, Quantitative Polymerase Chain Reaction; GSH, Reduced Glutathione; GSSG, Oxidized Glutathione; MDA, Malondialdehyde; ATP7B, ATPase Copper Transporting Beta; COMMD1, Copper Metabolism MURR1 Domain‐containing protein 1; RBX1, RING Box Protein 1; IF, Immunofluorescence; SD, Standard Deviation; ANOVA, one‐way analysis of variance.

**FIGURE 4:**
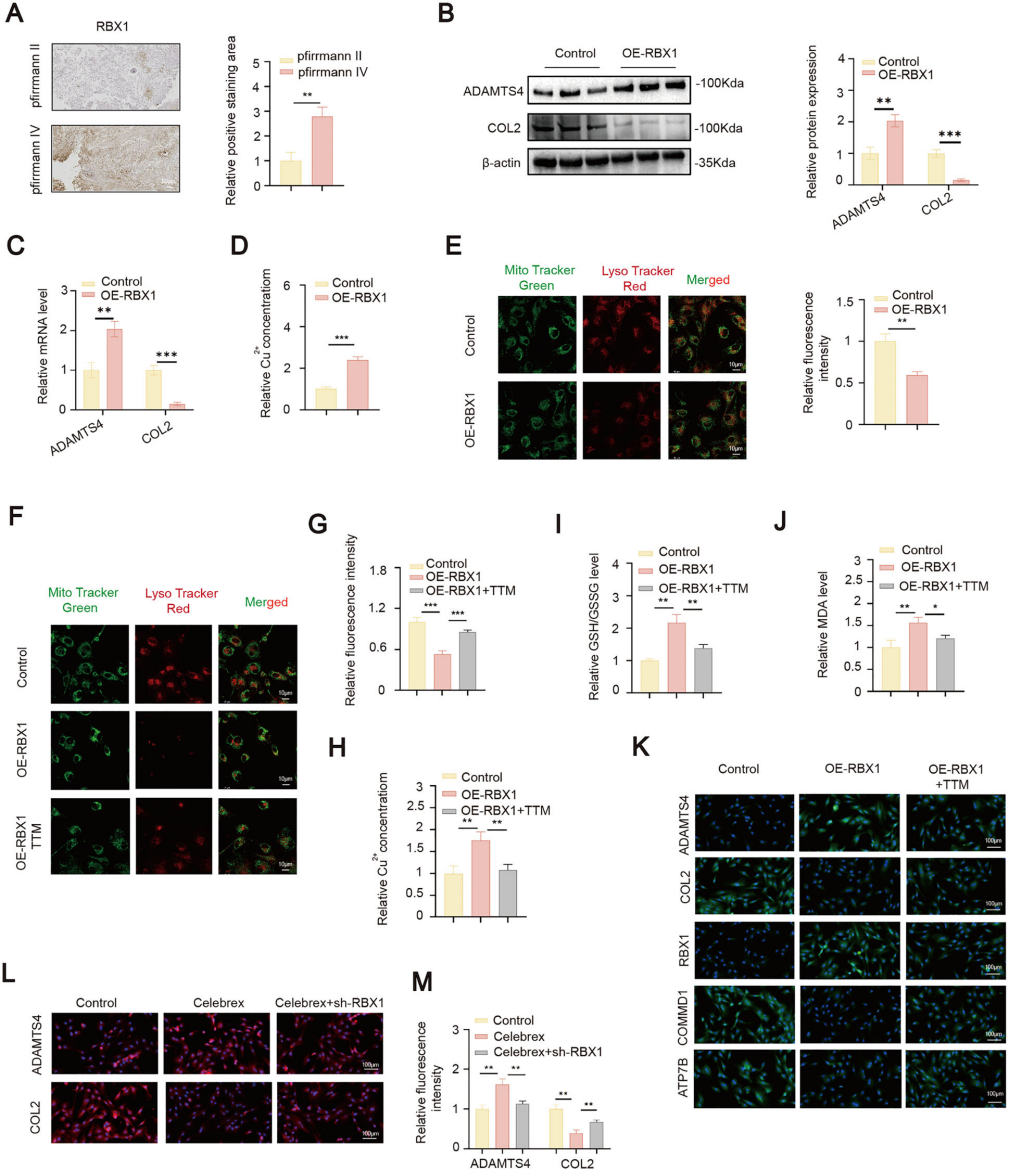
RBX1 drives NP cell degeneration via cuproptosis. (A) Representative IHC staining images and semi‐quantitative analysis of RBX1 expression in human NP tissues section with different degeneration degrees (*n* = 3). (B,C) In vitro analysis of related (ADAMTS4 and COL2) protein levels by Wb (B) and qPCR (C) in NP cells transfected as indicated. (D–J) The cuproptosis levels in NP cells transfected as indicated were determined by Cu^2+^ (D, H), GSH/GSSG (I), mitophagy (E, F, G), and MDA (J) levels. (K) In vitro analysis of related (ADAMTS4 and COL2), cuproptosis (ATP7B and COMMD1), HSP90, and RBX1 levels were determined by cellular IF staining of RBX1 overexpression with or without TTM‐treated NP cells. (L, M) In vitro analysis of related (ADAMTS4 and COL2) protein levels was determined by cellular IF staining of Celebrex with or without shRBX1‐treated NP cells. All data are presented as mean ± SD. Comparisons between two groups were performed using an unpaired two‐tailed Student's *t*‐test or ANOVA followed by Tukey's post hoc test. A *p*‐value less than 0.05 was considered statistically significant. ^*^ indicates *p* < 0.05, ^**^ indicates *p* < 0.01, ^***^ indicates *p* < 0.001, while “ns” indicates a lack of statistical significance. NP, Nucleus Pulposus; IHC, Immunohistochemistry; RBX1, RING Box Protein 1; ADAMTS4, A Disintegrin and Metalloproteinase with Thrombospondin Motifs 4; COL2, Type II Collagen; Wb, Western blot; qPCR, Quantitative Polymerase Chain Reaction; GSH, Reduced Glutathione; GSSG, Oxidized Glutathione; MDA, Malondialdehyde; ATP7B, ATPase Copper Transporting Beta; COMMD1, Copper Metabolism MURR1 Domain‐containing protein 1; HSP90, Heat Shock Protein 90; TTM, Tetrathiomolybdate; IF, Immunofluorescence; SD, Standard Deviation; ANOVA, one‐way analysis of variance.

We first validated the clinical relevance of this pathway in human intervertebral disc (IVD) tissue samples. Immunohistochemistry (IHC) analysis revealed that the protein expression levels of both HSP90 and RBX1 were significantly elevated in severely degenerated NP tissues compared to mildly degenerated tissues (Figures 3A and 4A). This finding suggests their potential important roles in the pathological process of IDD. Subsequently, we validated their pathogenic roles through in vitro gain‐of‐function and loss‐of‐function assays. The overexpression of either HSP90 or RBX1 in NP cells markedly exacerbated the imbalance in ECM metabolism. This was specifically evidenced by a significant upregulation in the mRNA and protein levels of the catabolic enzyme ADAMTS4, alongside a pronounced inhibition of the anabolic marker COL2 expression (refer to Figures 3B and 4B,C Figure S3A). More importantly, overexpression of either protein was sufficient to successfully induce a series of typical cuproptosis phenotypes, including diminished intracellular mitophagy, abnormal accumulation of copper ions, an increased GSH/GSSG, and elevated levels of the lipid peroxidation product MDA (Figures 3C–F and 4D,E; Figure S4A,B). To confirm that cuproptosis is the downstream executor, we performed rescue experiments using the cuproptosis‐specific inhibitor TTM. The findings demonstrated that TTM treatment effectively reversed all cuproptosis‐related phenotypes induced by the overexpression of HSP90 or RBX1 and partially ameliorated the ECM metabolic disorder (refer to Figures 3G–I and 4F–K; Figures S3B–D and S4C). This provides direct evidence that the activation of cuproptosis constitutes the central mechanism through which HSP90 and RBX1 exacerbate the degeneration of NP cells. Furthermore, functional rescue experiments indicated that knocking down HSP90 or RBX1 could partially rescue the ECM metabolic imbalance, the upregulation of RBX1 protein levels, and the downregulation of the key copper efflux proteins COMMD1 and ATP7B caused by high concentrations of celecoxib (Figures 3J–L and 4L,M), thereby establishing the critical role of this signaling axis in mediating celecoxib toxicity.

**FIGURE 5:**
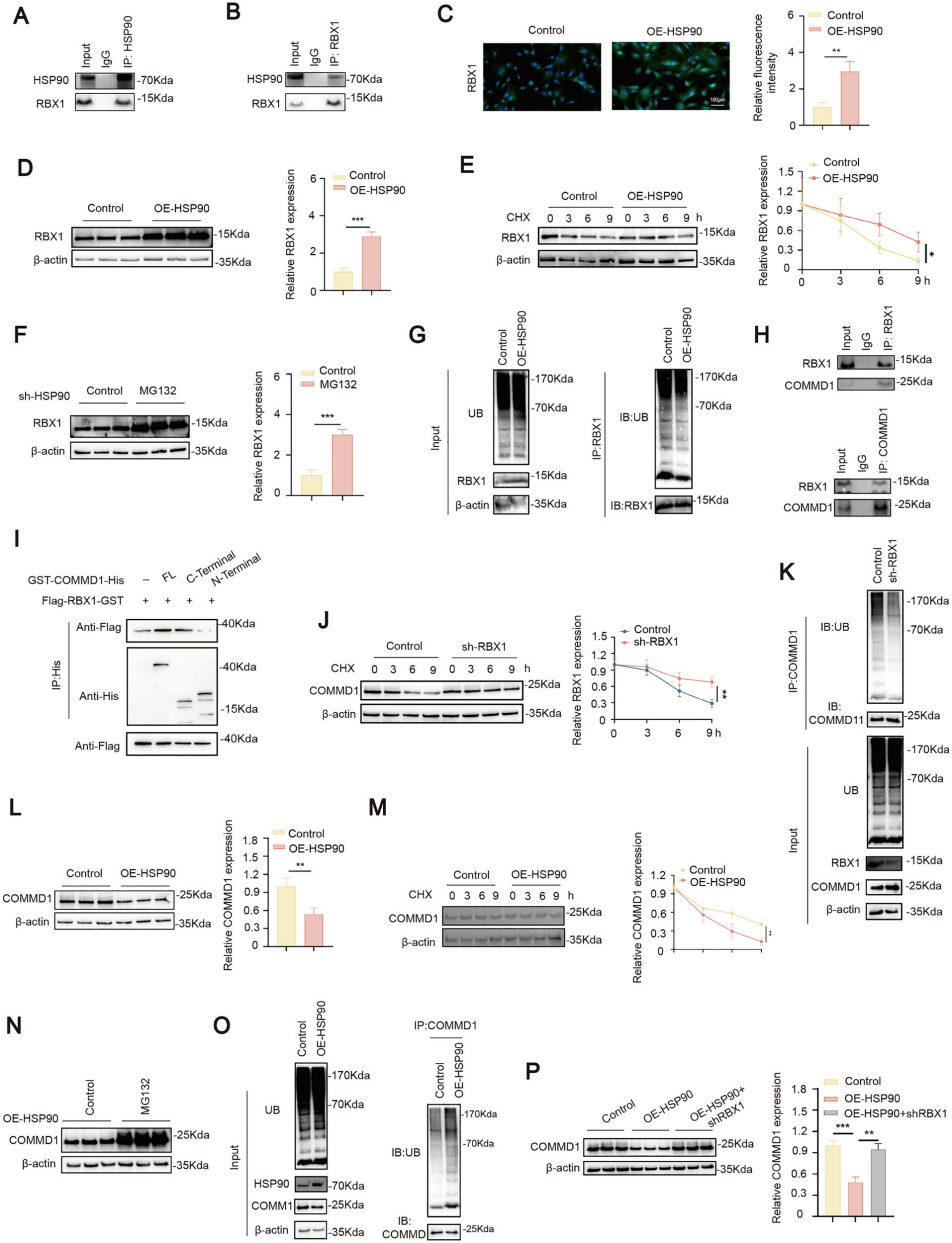
Unraveling the HSP90‐RBX1‐COMMD1 regulatory axis. (A, B) Cell lysates of NP cells were immunoprecipitated with IgG, HSP90(A) or RBX1(B) antibodies, and IB assays were performed using HSP90 and RBX1 antibodies. (C, D) In vitro analysis of RBX1 levels were determined by cellular IF staining (C) and Wb (D) of HSP90‐overexpression NP cells. (E) NP cells were transfected with Control or HSP90‐overexpression plasmid, and then treated with CHX (50 µg/mL) and collected at the indicated times. Cell lysates were subjected to Wb with antibodies against RBX1. (F) NP cells were transfected with HSP90 shRNA and treated with MG132 (20 µm for 8 h) before collecting. Cell lysates were subjected to Wb. (G) NP cells were transfected with HSP90 and treated with MG132 (20 µm for 8 h) before collecting. Cell lysates were subjected to d‐IP and Wb assays. (H) Cell lysates of NP cells were immunoprecipitated with IgG, RBX1 or COMMD1 antibodies, and IB assays were performed using indicated antibodies. (I) NP cells were co‐transfected with His‐COMMD1 WT and Flag‐RBX1 WT or domain overexpression plasmid. Cell lysates were subjected to IP, followed by Wb with antibodies against His and Flag. (J) NP cells were transfected with Control or RBX1‐deletion plasmid, and then treated with CHX (50 µg/mL) and collected at the indicated times. Cell lysates were subjected to Wb with antibodies against COMMD1. (K) NP cells were transfected with sh‐RBX1 and treated with MG132 (20 µm for 8 h) before collecting. Cell lysates were subjected to d‐IP and Wb assays. (L) In vitro analysis of COMMD1 levels were determined by cellular Wb of HSP90‐overexpression NP cells. (M) NP cells were transfected with Control or HSP90‐overexpression plasmid, and then treated with CHX (50 µg/mL) and collected at the indicated times. Cell lysates were subjected to Wb with antibodies against COMMD1. (N) HSP90‐overexpression NP cells and treated with MG132 (20 µm for 8 h) before collecting. Cell lysates were subjected to Wb. (O) NP cells were transfected with HSP90‐overexpressing plasmid and treated with MG132 (20 µm for 8 h) before collecting. Cell lysates were subjected to d‐IP and Wb assays. (P) In vitro analysis of COMMD1 levels were determined by cellular Wb of NP cells treated as indicated. All data are presented as mean ± SD. Comparisons between two groups were performed using an unpaired two‐tailed Student's *t*‐test or ANOVA followed by Tukey's post hoc test. A *p*‐value less than 0.05 was considered statistically significant. ^*^ indicates *p* < 0.05, ^**^ indicates *p* < 0.01, ^***^ indicates *p* < 0.001, while “ns” indicates a lack of statistical significance. NP, Nucleus Pulposus; IgG, Immunoglobulin G; IB, Immunoblot; HSP90, Heat Shock Protein 90; RBX1, RING Box Protein 1; IF, Immunofluorescence; Wb, Western blot; CHX, Cycloheximide; IP, Immunoprecipitation; COMMD1, Copper Metabolism MURR1 Domain‐containing protein 1; qPCR, Quantitative Polymerase Chain Reaction; SD, Standard Deviation; ANOVA, one‐way analysis of variance.

Next, we aimed to uncover the upstream molecular mechanism by which HSP90 regulates RBX1. Co‐Immunoprecipitation (Co‐IP) assays substantiated a direct physical interaction between HSP90 and RBX1 in NP cells (Figure 5A,B). In cells overexpressing HSP90, a notable elevation in RBX1 protein levels was observed, whereas its mRNA levels remained unchanged (Figure 5C,D; Figure S5A), indicating post‐translational regulation. Considering the established role of HSP90 as a molecular chaperone in preserving the stability of client proteins, we postulate that HSP90 may influence the turnover of RBX1 protein. Cycloheximide (CHX) chase assays demonstrated that HSP90 overexpression significantly slowed the degradation rate of the RBX1 protein and extended its half‐life (Figure 5E). Further experiments using MG132 revealed that in HSP90‐knockdown cells, the RBX1 protein accumulation effect induced by MG132 was more pronounced (Figure 5F), suggesting that more RBX1 is degraded via the ubiquitin‐proteasome pathway in the absence of HSP90. To obtain direct evidence, we performed in vivo ubiquitination assays. The findings indicated that in cells overexpressing HSP90, the signal intensity of ubiquitin chains associated with RBX1 was markedly reduced compared to the control group (Figure 5G). Collectively, these data substantiate the conclusion that HSP90 exerts a positive regulatory effect on the stabilization of the RBX1 protein at the post‐translational level by inhibiting its ubiquitination and subsequent proteasomal degradation.

Having clarified the upstream regulation, we further investigated the downstream targets of RBX1. Co‐IP experiments found that RBX1 could bind to COMMD1, an important intracellular copper efflux regulatory protein (Figure 5H). Through domain mapping analysis, we determined that the N‐terminus of RBX1 and the C‐terminus of COMMD1 are required for their interaction (Figure 5I; Figure S5B). Functionally, in RBX1‐overexpressing NP cells, the protein level of COMMD1 was significantly downregulated, while its mRNA level remained stable (Figure S5C–E), again pointing to a post‐translational degradation mechanism. CHX chase assays demonstrated that the knockdown of RBX1 substantially delayed the degradation of the COMMD1 protein (Figure 5J). Further in vivo ubiquitination assays offered direct evidence, revealing a significant reduction in the polyubiquitination levels of COMMD1 in RBX1‐knockdown cells (Figure 5K). Collectively, these findings elucidate the role of RBX1 as an E3 ubiquitin ligase that facilitates the ubiquitination and subsequent proteasomal degradation of COMMD1. In a corresponding manner, the overexpression of HSP90 results in the downregulation of COMMD1 protein levels by promoting its ubiquitination and subsequent degradation, without altering its mRNA levels, through the modulation of RBX1 (refer to Figure 5L–P; Figure S5F,G). Furthermore, our validation experiments demonstrate that the knockdown of COMMD1 independently influences the degeneration of NP cells, autophagy, and oxidative stress by modulating cuproptosis (Figure S5H–P).

**FIGURE 6:**
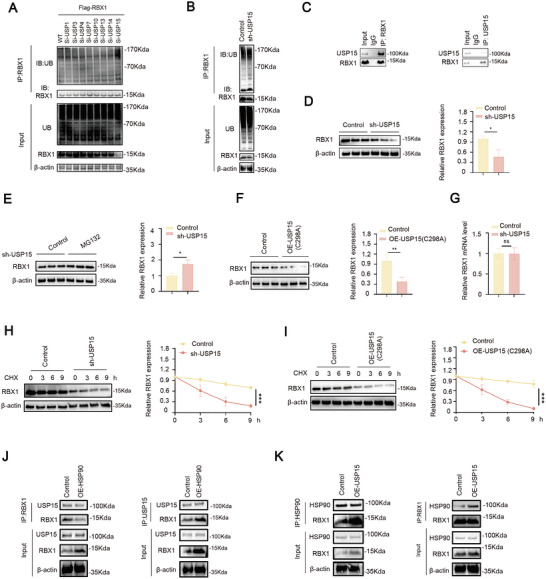
USP15 acts as the deubiquitinase of RBX1 and forms a reciprocal enhancement loop with HSP90 for RBX1 binding. (A) NP cells were co‐transfected with Flag‐RBX1 or different siDUBs and treated with MG132 (20 µm for 8 h) before collecting. Cell lysates were subjected to d‐IP and Wb assays. (B) NP cells were transfected with USP15 shRNA and treated with MG132 (20 µm for 8 h) before collecting. Cell lysates were subjected to Wb. (C) Cell lysates of NP cells were immunoprecipitated with IgG, RBX1 or USP15 antibodies, and IB assays were performed using RBX1 and USP15 antibodies. (D) In vitro analysis of RBX1 levels were determined by Wb of USP15‐deletion NP cells. (E) NP cells were transfected with USP15 shRNA and treated with MG132 (20 µm for 8 h) before collecting. Cell lysates were subjected to Wb. (F) NP cells transfected with USP15‐C298A and subjected to Wb with antibodies against RBX1. (G) In vitro analysis of RBX1 levels were determined by qPCR of USP15‐deletion NP cells. (H) NP cells were transfected with Control or USP15‐deletion plasmid, and then treated with CHX (50 µg/mL) and collected at the indicated times. Cell lysates were subjected to Wb with antibodies against RBX1. (I) NP cells were transfected with Control or USP15‐C298A plasmid, and then treated with CHX (50 µg/mL) and collected at the indicated times. Cell lysates were subjected to Wb with antibodies against RBX1. (J) Cell lysates of control and HSP90‐overexpression NP cells were immunoprecipitated with RBX1 or USP15 antibodies, and IB assays were performed using RBX1 and USP15 antibodies. (K) Cell lysates of control and USP15‐overexpression NP cells were immunoprecipitated with RBX1 or HSP90 antibodies, and IB assays were performed using RBX1 and HSP90 antibodies. All data are presented as mean ± SD. Comparisons between two groups were performed using an unpaired two‐tailed Student's *t*‐test or ANOVA followed by Tukey's post hoc test. A *p*‐value less than 0.05 was considered statistically significant. ^*^ indicates *p* < 0.05, ^**^ indicates *p* < 0.01, ^***^ indicates *p* < 0.001, while “ns” indicates a lack of statistical significance. USP15, Ubiquitin Specific Peptidase 15; RBX1, RING Box Protein 1; HSP90, Heat Shock Protein 90; NP, Nucleus Pulposus; MG132, Carbobenzoxy‐Leu‐Leu‐leucinal; IP, Immunoprecipitation; Wb, Western blot; IgG, Immunoglobulin G; IB, Immunoblot; qPCR, Quantitative Polymerase Chain Reaction; CHX, Cycloheximide; SD, Standard Deviation; ANOVA, one‐way analysis of variance.

To complete the regulatory network of RBX1 proteostasis, we explored its deubiquitination mechanism. By screening deubiquitinases associated with HSP90, we found that knockdown of USP15 most potently enhanced RBX1 ubiquitination and reduced its protein abundance (Figure 6A,B). USP15 directly interacts with RBX1 (Figure 6C), and its catalytically inactive mutant (USP15 C298A) failed to stabilize RBX1 (Figure 6F,I). A series of experiments demonstrated that USP15 deubiquitinates and stabilizes the RBX1 protein (Figure 6D,E,G,H). Notably, the overexpression of HSP90 significantly enhanced the interaction between USP15 and RBX1 (Figure 6J). Conversely, elevated levels of USP15 also promoted the interaction between HSP90 and RBX1 (Figure 6K). These findings suggest that HSP90 may function as a molecular scaffold, facilitating the deubiquitination of RBX1 by USP15. Together, USP15 and HSP90 synergistically contribute to maintaining the elevated protein levels and stability of RBX1.

**FIGURE 7:**
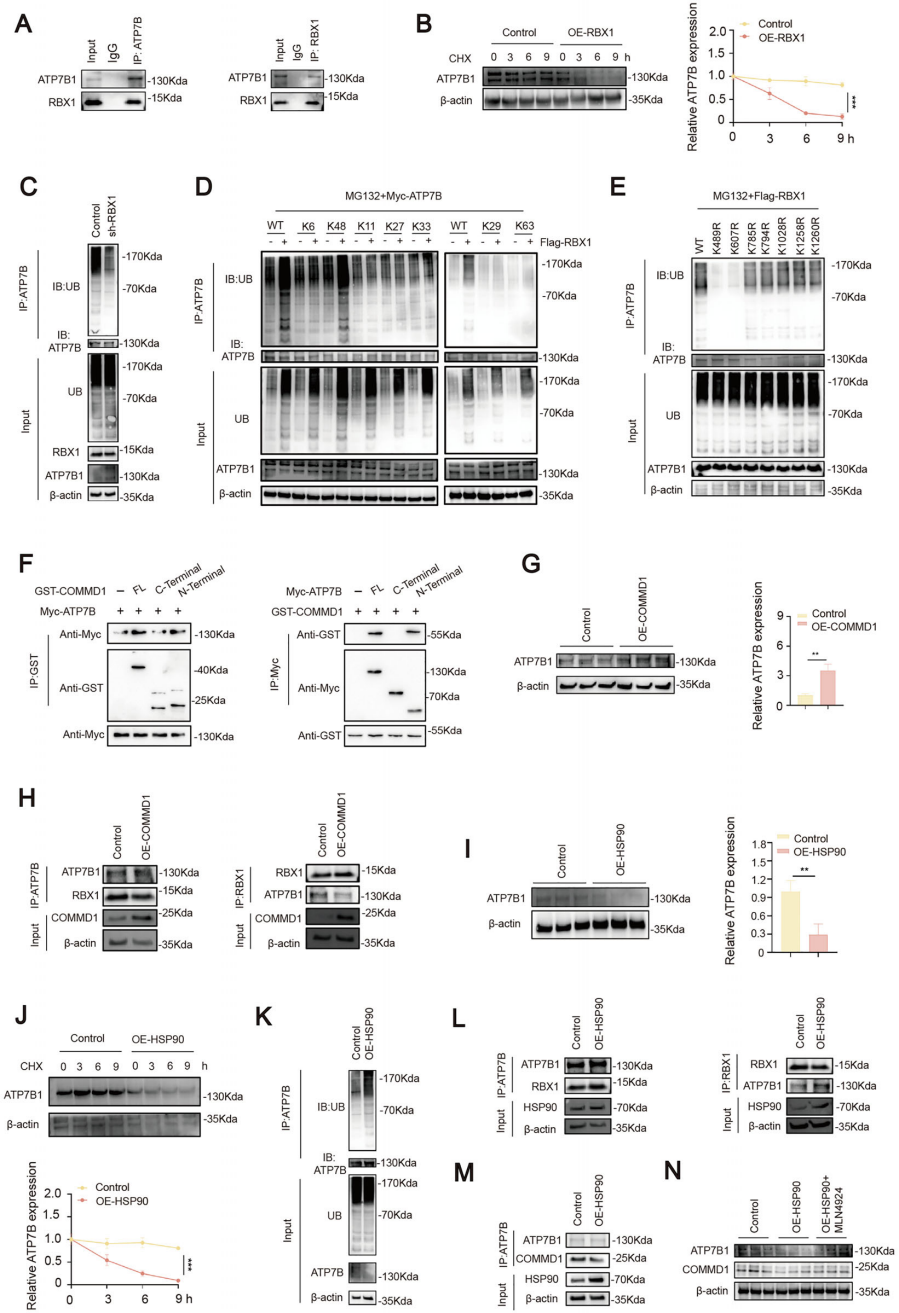
HSP90, RBX1, and COMMD1 cooperatively regulate ATP7B K48‐linked polyubiquitination and stabilization. (A) Cell lysates of NP cells were immunoprecipitated with IgG, ATP7B or RBX1 antibodies, and IB assays were performed using ATP7B and RBX1 antibodies. (B) NP cells were transfected with Control or RBX1‐overexpressing plasmid, and then treated with CHX (50 µg/mL) and collected at the indicated times. Cell lysates were subjected to Wb with antibodies against ATP7B. (C) NP cells were transfected with RBX1 shRNA and treated with MG132 (20 µm for 8 h) before collecting. Cell lysates were subjected to d‐IP. (D) NP cells were co‐transfected with Myc‐ATP7B, Flag‐RBX1, WT Ubiquitin or different ubiquitin overexpression plasmid and treated with MG132 (20 µm for 8 h) before collecting. Cell lysates were subjected to d‐IP and Wb assays. (E) NP cells transfected with Flag‐RBX1, ubiquitin K48 and different Myc‐ATP7B mutants were subjected to d‐IP with ant‐Myc antibody and analyzed by Wb. Cells were treated with MG132 (20 µm) for 8 h before collection. (F) NP cells were co‐transfected with GST‐COMMD1 WT and Myc‐COMMD1 WT or domain overexpression plasmid. Cell lysates were subjected to IP, followed by Wb with antibodies against GST and Myc. (G) In vitro analysis of ATP7B levels were determined by Wb of COMMD1‐overexpressing NP cells. (H) Cell lysates of control and COMMD1‐overexpression NP cells were immunoprecipitated with ATP7B or RBX1 antibodies, and IB assays were performed using ATP7B and RBX1 antibodies. (I) In vitro analysis of ATP7B levels were determined by Wb of HSP90‐overexpressing NP cells. (J) NP cells were transfected with Control or HSP90‐overexpressing plasmid, and then treated with CHX (50 µg/mL) and collected at the indicated times. Cell lysates were subjected to Wb with antibodies against ATP7B. (K) HSP90‐control and overexpression NP cells were treated with MG132 (20 µm for 8 h) before collecting. Cell lysates were subjected to d‐IP. (L) Cell lysates of control and HSP90‐overexpression NP cells were immunoprecipitated with ATP7B or RBX1 antibodies, and IB assays were performed using ATP7B and RBX1 antibodies. (M) Cell lysates of control and HSP90‐overexpression NP cells were immunoprecipitated with ATP7B antibody, and IB assays were performed using ATP7B and COMMD1 antibodies. (N) In vitro analysis of ATP7B and COMMD1 levels was determined by Wb of HSP90 overexpression with or without MLN4924‐treated NP cells. All data are presented as mean ± SD. Comparisons between two groups were performed using an unpaired two‐tailed Student's *t*‐test or ANOVA followed by Tukey's post hoc test. A *p*‐value less than 0.05 was considered statistically significant. ^*^ indicates *p* < 0.05, ^**^ indicates *p* < 0.01, ^***^ indicates *p* < 0.001, while “ns” indicates a lack of statistical significance. HSP90, Heat Shock Protein 90; RBX1, RING Box Protein 1; COMMD1, Copper Metabolism MURR1 Domain‐containing protein 1; ATP7B, ATPase Copper Transporting Beta; NP, Nucleus Pulposus; IgG, Immunoglobulin G; IB, Immunoblot; CHX, Cycloheximide; Wb, Western blot; MG132, Carbobenzoxy‐Leu‐Leu‐leucinal; IP, Immunoprecipitation; SD, Standard Deviation; ANOVA, one‐way analysis of variance.

We further extended our investigation to ATP7B, another key copper transporter located on the cell membrane. Results showed that RBX1 could also interact with ATP7B (Figure 7A) and mediate its K48‐linked polyubiquitination and degradation, with the main ubiquitination sites identified as K489 and K607 (Figure 7C–E). CHX assays indicated that RBX1 overexpression significantly shortened the half‐life of the ATP7B protein without affecting its mRNA level (Figure 7B; Figure S6B). The knockdown of RBX1 resulted in an opposing effect (Figure S6A). Notably, our findings indicate that COMMD1 can directly bind to ATP7B, with its interaction domain precisely encompassing the ubiquitination site region on ATP7B targeted by RBX1 (Figure 7F). From a functional perspective, the overexpression of COMMD1 was observed to elevate ATP7B protein levels, delay its degradation, and reduce its ubiquitination (Figure 7G; Figure S6C,D). Competitive Co‐IP experiments revealed an intricate regulatory relationship: COMMD1 can competitively bind to ATP7B against RBX1 (Figure 7H). This implies that under normal physiological conditions, COMMD1 acts like a “molecular shield,” physically hindering RBX1's access to and degradation of ATP7B through their binding.

Under celecoxib stress, the activation of upstream HSP90 disrupts this balance. We found that upregulation of HSP90 decreased the steady‐state level of ATP7B and accelerated its ubiquitination‐dependent degradation (Figure 7I–K). Mechanistically, while stabilizing RBX1, HSP90, on one hand, enhanced the interaction between RBX1 and ATP7B (Figure 7L), and on the other hand, suppressed the binding between COMMD1 and ATP7B (Figure 7M; Figure S6E). This weakened the protective effect of COMMD1 on ATP7B, making ATP7B more susceptible to targeting and degradation by the active RBX1. Upon employing the CRL complex‐specific inhibitor MLN4924 to suppress RBX1 activity, our observations indicated that, within the framework of HSP90 overexpression, the restoration of ATP7B protein levels was significantly more pronounced compared to that of COMMD1 (Figure 7N). This finding strongly implies that within this pathway, ATP7B serves as the principal degradation target of RBX1, whereas the degradation of COMMD1 might represent a secondary occurrence or function as part of a negative feedback mechanism. Therefore, under conditions of excessive HSP90 activation, the accumulation and hyperactivity of RBX1 ultimately lead to the depletion of both key copper efflux proteins, COMMD1 and ATP7B, causing a complete collapse of intracellular copper ion homeostasis and thereby triggering cuproptosis.

### 2.4 In Vivo Inhibition of HSP90 or Cuproptosis Reverses IDD Exacerbated by High Concentrations of Celecoxib

**FIGURE 8:**
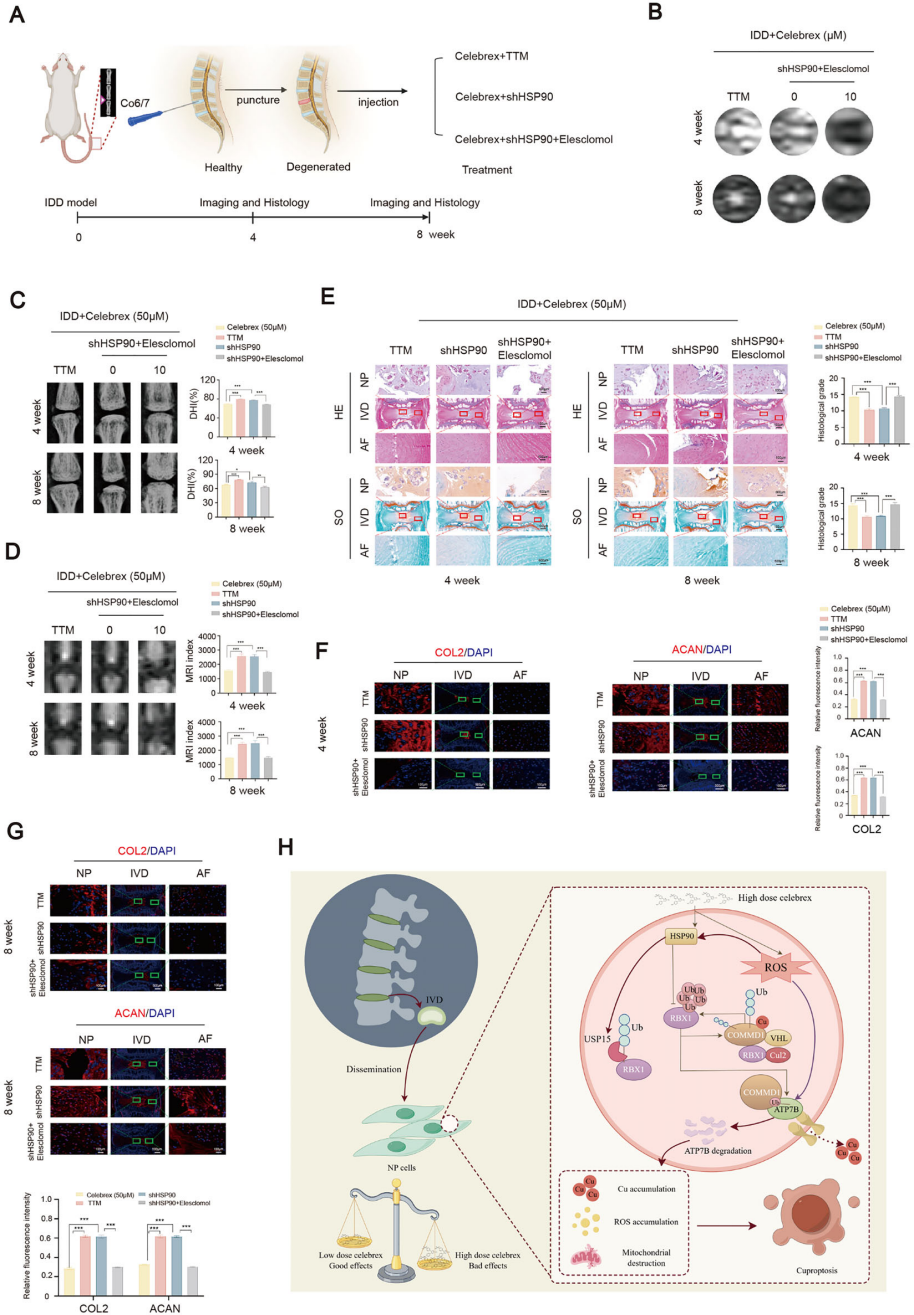
In vivo inhibition of HSP90 or cuproptosis reverses IDD exacerbated by high‐concentration celecoxib.​​ (A) Schematic diagram of the animal experiment. (B) Representative MRI cross‐sectional scans obtained at 4‐ and 8‐weeks post‐intervention (*n* = 6). (C) Representative X‐ray scans and DHI index of IVDs at 4‐ and 8‐weeks post‐intervention (*n* = 6). (D) Representative MRI scans and their MRI index calculations obtained at 4‐ and 8‐weeks post‐intervention (*n* = 6). (E) Representative HE staining and SO staining images at 4‐ and 8‐weeks post‐intervention (*n* = 6). (F, G) COL2 and ACAN IF staining detection and quantitative analysis of IF staining at 4‐ and 8‐weeks post‐intervention (*n* = 6). (H) Schematic diagram of the biological mechanism. All data are presented as mean ± SD. Comparisons between two groups were performed using an unpaired two‐tailed Student's *t*‐test or ANOVA followed by Tukey's post hoc test. A *p*‐value less than 0.05 was considered statistically significant. ^*^ indicates *p* < 0.05, ^**^ indicates *p* < 0.01, ^***^ indicates *p* < 0.001, while “ns” indicates a lack of statistical significance. HSP90, Heat Shock Protein 90; IDD, Intervertebral Disc Degeneration; MRI, Magnetic Resonance Imaging; DHI, Disc Height Index; IVD, Intervertebral Disc; HE, Hematoxylin and Eosin; SO, Safranin O; COL2, Type II Collagen; ACAN, Aggrecan; IF, Immunofluorescence; SD, Standard Deviation; ANOVA, one‐way analysis of variance.

To validate in vivo the hypothesis that high concentrations of celecoxib exacerbate IDD through the HSP90‐cuproptosis pathway, we designed a series of in vivo animal rescue experiments (schematic diagram of the experimental procedure is shown in Figure 8A). After needle puncture modeling in rats, they were randomly divided into the following four groups for intradiscal injection treatment: 1. IDD + 50 µm Celecoxib group; 2. IDD + 50 µm Celecoxib + TTM group; 3. IDD + 50 µm Celecoxib + sh‐HSP90 group; 4. IDD + 50 µm Celecoxib + sh‐HSP90 + Elesclomol group. Imaging assessments indicated that inhibiting HSP90 or cuproptosis can rescue the negative effects of high‐concentration celecoxib (Figure 8B–D). In the high‐concentration celecoxib group, the DHI and MRI indices of the IVD were significantly diminished, exhibiting no notable difference from the prior IDD group (refer to section 3.1). This suggests that a concentration of 50 µm celecoxib was ineffective in ameliorating degeneration and may have perpetuated the degenerative condition to some degree. Conversely, both the TTM group and the sh‐HSP90 group demonstrated significant enhancements in DHI and MRI indices, with values markedly exceeding those observed in the high‐concentration celecoxib group. Histological sections (Figure 8E) revealed that the IVD structure in these two groups was markedly protected, with more retention of gel‐like ECM in the NP, a more intact AF structure, and broader SO‐positive staining areas. Molecular biology detection further supported the aforementioned morphological improvements. IF staining of tissue samples revealed that, relative to the high‐concentration celecoxib group, the fluorescence signals of COL2 and ACAN were markedly intensified in the TTM and sh‐HSP90 groups (Figure 8F,G). Consequently, we conclude that the therapeutic efficacy of celecoxib in IDD is limited to a specific concentration range, beyond which its effectiveness diminishes, and it may induce toxic effects. Low concentrations (≤20 µm) exert protective effects through mechanisms such as anti‐inflammation; whereas concentrations exceeding this window (>20 µm, e.g., 50 µm) activate the HSP90/RBX1/COMMD1‐ATP7B axis, induce cuproptosis, thereby counteracting its therapeutic effect or even exacerbating damage, as shown in the mechanism diagram (Figure 8H).

## 3 Discussion

Given the widespread clinical use of NSAIDs, the unexpected link between drug exposure and copper‐dependent cell death revealed in this study carries significant broad implications. These findings extend the emerging concept of cuproptosis into the field of musculoskeletal degenerative diseases and highlight copper homeostasis as a previously underappreciated determinant of drug toxicity.​ Through a series of systematic in vivo and in vitro experiments, this study clarifies the unprecedented biphasic, concentration‐dependent effect of celecoxib in IDD and its narrow therapeutic window.​ This study identified a narrow effective concentration window for celecoxib in the treatment of IDD, with 20 µm serving as the critical threshold. At this concentration, celecoxib most effectively inhibits inflammation and protects the ECM at the cellular level, while optimally preserving disc structure and composition in animal models. However, once the concentration exceeds 20 µm, its protective effects are reversed, and a cuproptosis toxicity pathway characterized by disruption of copper homeostasis (copper accumulation), inhibition of mitophagy, and lipid peroxidation (elevated MDA levels) is activated, thereby exacerbating degeneration. More importantly, we elucidate, for the first time, a comprehensive signaling axis wherein the molecular chaperone HSP90 activates the E3 ubiquitin ligase RBX1, ultimately leading to the induction of cuproptosis‐a novel form of cell death. Our study yields several key first demonstrations: (1) supratherapeutic concentrations of celecoxib directly trigger cuproptosis in NP cells, revealing that NSAID‐induced toxicity can be mediated through copper‐handling pathways; (2) HSP90 acts as the critical molecular switch converting celecoxib from a therapeutic agent into a pro‐degenerative one; (3) RBX1 drives cuproptosis in disc tissue by mediating K48‐linked polyubiquitination of ATP7B.

The principal outcome of this study is the identification of a precise concentration threshold that distinguishes the therapeutic benefits from the toxic effects of celecoxib. At lower concentrations (10–20 µm), our findings align with a substantial body of previous research, demonstrating that celecoxib effectively reduces IL‐1β‐induced levels of pro‐inflammatory mediators, including TNF‐α and IL‐6, via its well‐documented mechanism of COX‐2 inhibition [29]. Importantly, we observed that this anti‐inflammatory effect coincided with an enhancement in ECM metabolism, specifically by promoting the synthesis of COL2 and ACAN while suppressing the expression of catabolic enzymes such as ADAMTS4. At this concentration, celecoxib demonstrated distinct cytoprotective and ECM‐protective effects, consistent with the documented chondroprotective potential of NSAIDs in osteoarthritis [30, 31]. These findings support the hypothesis that the judicious use of low‐concentration celecoxib may be advantageous in delaying the progression of IDD under certain conditions. Upon surpassing a concentration of 20 µm and reaching levels between 30 and 50 µm, a notable paradigm shift was observed: the protective effect of celecoxib not only dissipated but also began to exacerbate the degenerative phenotype. This phenomenon cannot be solely attributed to reductions in cell viability, as cellular activity did not exhibit significant diminishment even at concentrations of 30–40 µm. Nevertheless, beneficial biological functions, such as ECM synthesis, had already commenced a decline. We refer to this as “functional toxicity,” which fundamentally differs from the traditional linear dose‐toxicity model. This strongly suggests that at elevated concentrations, a predominant toxic mechanism, independent of COX‐2 inhibition, is activated. Indeed, previous studies have suggested that high concentrations of NSAIDs might induce apoptosis, autophagy, or endoplasmic reticulum stress through COX‐independent pathways [32, 33, 34, 35, 36, 37]. For example, NSAIDs, including celecoxib, have been reported to inhibit the Protein Kinase B (Akt) signaling pathway and activate caspase [34, 38, 39, 40].

HSP90, a pivotal molecular chaperone, serves as a central regulator of the cellular response to diverse proteotoxic stresses. In degenerative joint disorders, including osteoarthritis, there is an upregulation of HSP90 expression, which exacerbates the pathological process by stabilizing critical proteins involved in inflammatory signaling pathways, such as the inhibitor of nuclear factor kappa B kinase subunit beta (IKKβ) [41, 42, 43]. Our research demonstrated a significant elevation of HSP90 expression in degenerated human and rat NP tissues, with a positive correlation to the severity of degeneration, thereby extending its implicated role in IDD. Notably, our findings indicate that elevated concentrations of celecoxib act as a significant inducer of HSP90 expression in NP cells. This observation suggests that the upregulation of HSP90 may represent a cellular response to high‐concentration drug stress, potentially involving oxidative stress or mitochondrial dysfunction; however, this stress response ultimately contributes to a deleterious cycle [44, 45]. HSP90 plays a crucial role in regulating the stability of various signaling proteins and E3 ubiquitin ligases. In our research, we identified RBX1 as a significant downstream target of HSP90, particularly in the context of mediating celecoxib‐induced toxicity. RBX1 functions as the catalytic core of CRL complexes, which are pivotal in the ubiquitination of substrate proteins, thereby affecting their stability, activity, or localization. Through CHX chase assays and ubiquitination analyses, we demonstrated that HSP90 significantly enhances the stability of RBX1 by inhibiting its ubiquitination and subsequent degradation.

Intracellular copper ion concentration is meticulously regulated through a highly dynamic balance. This homeostasis is achieved via a coordinated system encompassing import, chelation, storage, and export mechanisms [46]. The export system constitutes the primary mechanism for preventing copper accumulation and is chiefly facilitated by two categories of proteins. The first category comprises members of the P1B‐type ATPase family, specifically ATP7A and ATP7B, which harness the energy from ATP hydrolysis to transport Cu^2^^+^ either out of the cell or into secretory pathways. The second category includes the CCC complex (COMMD/CCDC22/CCDC93), featuring the COMMD1 protein. This complex indirectly influences copper homeostasis by regulating the endocytosis and recycling of various membrane proteins, including copper transporters. A pivotal finding of our study is the elucidation of RBX1's remarkably effective dual‐strike strategy, which synergistically disrupts two essential copper export systems. Initially, through in vivo ubiquitination assays and point mutation validation, we established that RBX1 directly facilitates the K48‐linked polyubiquitination of ATP7B at key sites (K489, K607), resulting in its degradation by the 26S proteasome. This action effectively halts the cell's primary active copper transport mechanism. However, the complexity of this mechanism extends beyond this initial step. Our research demonstrates that RBX1 concurrently targets COMMD1 for degradation, leading to intricate cascade effects. Notably, we discovered that COMMD1 is a crucial regulator necessary for the optimal activity of the Cullin‐RING E3 Ub ligase complex, of which RBX1 is a component. This implies that COMMD1 partially “activates” RBX1, enhancing its ability to target substrates such as ATP7B. Concurrently, COMMD1 itself is a degradation target of RBX1, establishing a classic “negative feedback loop.” Under physiological conditions, this “activate‐and‐degrade” mechanism may function to rapidly terminate signals, thereby preventing excessive activation of E3 ligase and exemplifying a sophisticated design for maintaining cellular homeostasis. However, in the pathological context characterized by sustained high concentrations of celecoxib and HSP90 activation, this delicate equilibrium is severely disrupted. The sustained overexpression of HSP90 leads to the aberrant stabilization and accumulation of RBX1, which in turn accelerates the degradation of COMMD1, culminating in severe consequences. Our study demonstrates that COMMD1 acts as a “molecular shield,” providing a protective effect on ATP7B. Through competitive binding assays and domain analysis, we determined that COMMD1 interacts with a specific region of the ATP7B protein, encompassing the ubiquitination sites targeted by RBX1. Consequently, the presence of COMMD1 induces steric hindrance, thereby obstructing RBX1's access to and interaction with ATP7B. One noteworthy observation is that following RBX1 inhibition, the restoration of ATP7B protein levels was significantly greater than that of COMMD1 (Figure 7N). This may suggest that under sustained high‐dose celecoxib exposure, ATP7B, as the key copper‐efflux pump, could be a more central target for maintaining copper homeostasis, and its degradation is a more direct driver triggering intracellular copper overload. In contrast, the degradation of COMMD1, on the one hand, may accelerate the destabilization of ATP7B, and on the other hand, by weakening its regulatory functions in other signaling pathways such as nuclear factor kappa‐light‐chain‐enhancer of activated B cells (NF‐κB), may synergistically exacerbate cellular inflammation and stress, thereby collectively driving the cells toward cuproptosis [47]. This difference in degradation dynamics and extent of recovery provides a new perspective for understanding the hierarchical contribution of different targets within this pathway.

While this study systematically elucidates the molecular mechanism by which high concentrations of celecoxib induce cuproptosis via the HSP90/RBX1 axis, several limitations should be acknowledged. First, the mechanistic investigation primarily relied on a rat NP cell model, and its generalizability to human cells requires further validation. Second, the relationship between the high drug concentrations used experimentally and clinical therapeutic doses must be interpreted with caution; their relevance in the local microenvironment of patients awaits confirmation through more in‐depth pharmacokinetic studies. Furthermore, the current findings are mainly derived from preclinical models, and there is a lack of prospective clinical data from patient cohorts for support. Third, this study established that upregulation of HSP90 serves as the critical “molecular switch” linking high concentrations of celecoxib to the RBX1/cuproptosis axis, while it did not elucidate the specific upstream signaling that induces HSP90 expression. This process may involve pathways such as drug‐induced oxidative stress, mitochondrial dysfunction, or the unfolded protein response [48], and its detailed mechanism awaits further investigation in future studies. Finally, the assessment of the cuproptosis pathway has room for expansion. Future studies could integrate more comprehensive detection methods (e.g., ultrastructural observation) and biomarkers to more fully delineate its activation state in IDD. The study confirmed cuproptosis by examining various phenotypes like mitophagy, redox status, copper buildup, and lipid peroxidation. Key molecular events involve copper ion‐induced oligomerization of proteins like ferredoxin 1 and dihydrolipoyl S‐acetyltransferase [49, 50]. Future research should directly observe this oligomerization using methods such as non‐reducing gel electrophoresis or Co‐IP. This would provide direct evidence for celecoxib‐induced cuproptosis, enhancing the understanding of the “HSP90‐RBX1‐copper homeostasis dysregulation” pathway identified in this study. These limitations point the way for subsequent research, including validation in models more closely resembling human physiology and exploration of the translational potential of this pathway.

Based on the aforementioned mechanisms, the findings of this study carry direct implications for clinical practice. This study suggests that exceeding a narrow intra‐discal celecoxib concentration window may inadvertently trigger cuproptosis and accelerate IDD. The actual local concentration of celecoxib in the IVD following conventional oral or injectable administration may accumulate and increase due to factors such as individual metabolic differences, local blood supply status, and long‐term use, potentially even reaching the threshold for triggering cuproptosis identified in this study. This suggests that drug exposure exceeding the safe therapeutic window, particularly at high local concentrations, may activate the HSP90/RBX1/cuproptosis axis, leading to a pro‐degenerative effect that contradicts its original anti‐inflammatory purpose. The mechanistic exploration in this study was primarily conducted in cell models under standard nutrient conditions. However, degenerated IVDs reside in a unique pathological microenvironment characterized by hypoxia, acidity, and nutrient deprivation [51, 52]. This hostile microenvironment itself can lead to cellular metabolic reprogramming, increased oxidative stress, and impaired proteasome function. It could imply that in the actual degenerative microenvironment, the concentration threshold (i.e., the therapeutic window) at which celecoxib induces toxicity might be narrower than what we observed under standard conditions. This primarily stems from the unique anatomical and physiological environment of the IVD. First, as an avascular tissue, the blood supply to the IVD‐especially the NP‐is extremely limited, which may lead to inefficient systemic drug clearance via the bloodstream, thereby prolonging the drug's local residence time. Second, the hypoxic, acidic, and low metabolic state of degenerated discs may further slow the rates of drug biotransformation and clearance. These factors collectively increase the risk of local drug accumulation exceeding the safe therapeutic window. Further research is necessary to investigate whether the sensitivity of NP cells to celecoxib is altered within this pathological context. Furthermore, the significance of the “HSP90‐RBX1‑cuproptosis” axis elucidated in this study may extend beyond celecoxib. Future research is warranted to investigate whether other NSAIDs or compounds with similar chemical structures or targets might also activate this shared pathway upon exceeding their specific concentration thresholds, thereby inducing similar concentration‑dependent toxicity in IVD tissues. Therefore, in the clinical management of chronic low back pain using NSAIDs, especially with long‐term or high‐dose regimens, it is crucial to carefully weigh the anti‐inflammatory benefits against the potential risk of accelerating tissue degeneration, and to emphasize the importance of maintaining drug concentrations within a defined therapeutic window. In the future, monitoring local drug concentrations in the disc or related biomarkers may provide guidance for personalized and safe medication use.

The determination of a 20 µm therapeutic threshold in our in vitro experiments significantly advances the development of localized drug delivery strategies for clinical application. In contrast, systemic administration of celecoxib yields disc concentrations that are considerably lower than this threshold, primarily due to the presence of the blood‐disc barrier. Consequently, the identification of this specific in vitro target supports the rationale for intradiscal delivery. As referenced, novel hydrogel‐based sustained‐release systems are being developed to achieve and maintain such localized therapeutic concentrations [53, 54, 55]. Hence, our findings offer a crucial pharmacological benchmark for the optimization of these injectable depot formulations, ensuring efficacy within the 10–20 µm therapeutic window while avoiding activation of the pro‐degenerative HSP90/RBX1/cuproptosis axis at elevated doses.

## 4 Methods

### 4.1 Clinical Sample Collection

The NP tissues utilized in this study were sourced from patient samples collected during spinal surgeries conducted within the department of spine surgery at our hospital. Specifically, degenerative NP tissues were procured from three patients who were clinically and radiographically diagnosed with IDD, while normal NP tissues were obtained from three patients without IDD who underwent surgical intervention due to acute vertebral fractures accompanied by neurological deficits. The severity of IDD was independently evaluated by three senior spinal surgeons using the Pfirrmann grading system based on preoperative MRI scans (comprehensive sample information is available in Table S1). The study protocol received approval from the Ethics Committee of East Hospital Affiliated to Tongji University (Approval No.: DF2025(CR)‐01 and TJBB05022101). Informed written consent was obtained from all patients prior to surgery, and the entire experimental procedure was conducted in strict accordance with the ethical principles outlined in the Declaration of Helsinki.

### 4.2 Cell Isolation, Culture, and Treatment

NP cells were isolated from one‐month‐old Sprague‐Dawley (SD) rats, procured from Shanghai SLAC Laboratory Animal Co., Ltd. The rats were euthanized via intraperitoneal administration of sodium pentobarbital at a dosage of 50 mg/kg. Subsequently, the lumbar segments were aseptically excised, and the IVD tissues were carefully dissected to isolate the NP regions. The NP tissue fragments underwent enzymatic digestion using a 0.1% collagenase type II solution (Sigma–Aldrich) for a duration of four hours in a constant temperature shaker set at 37°C. Following the cessation of digestion, the resultant cell suspension was filtered through a 200 µm cell strainer. The cells were then subjected to centrifugation, resuspended, and cultured in DMEM/F12 complete medium, which was supplemented with 10% fetal bovine serum (FBS, A5256701, Gibco) and 1% penicillin/streptomycin (15070063, Gibco). The cell cultures were maintained in an incubator at 37°C with 5% CO_2_ and saturated humidity, with the medium being refreshed every three days. All experimental procedures were conducted using passage 2 cells, which exhibited optimal growth characteristics and achieved 80%–90% confluence, thereby ensuring the stability and reproducibility of the experimental outcomes.

To replicate the inflammatory microenvironment characteristic of IVD degeneration, primary NP cells were exposed to 10 ng/mL of human recombinant IL‐1β (RIL1BI, Thermo Fisher) for a duration of 72 h without medium change, thereby establishing an in vitro model of IDD. Additional experimental conditions included transfection with short hairpin RNAs (shRNAs) targeting the genes HSP90, RBX1, and COMMD1, with the following sequences: shHSP90 (5′‐GGAACGTGATAAAGAAGTA‐3′), shRBX1 (5′‐CUGGGAUAUUGUGGUUGAUTT‐3′), and shCOMMD1 (5′‐AGCAGATCTTGAAGAAGCT‐3′). Other treatments involved the administration of Celecoxib (ab141988, Beyotime), the cuproptosis inducer Elesclomol (10 nM, HY‐12040, MedChemExpress), the cuproptosis inhibitor TTM (25 µm, HY‐W076067, MedChemExpress), the protein synthesis inhibitor CHX (50 µg/mL, 66‐81‐9, Sigma–Aldrich, with treatment duration varying according to the specific experimental protocol), and the proteasome inhibitor MG132 (20 µm, HY‐13259, MedChemExpress, pre‐treatment for 6 h). For gene silencing experiments, Lipofectamine 2000 (11668500, Invitrogen) was employed as the transfection reagent, in accordance with the manufacturer's instructions, to transfect 100 pmol of either siRNA or shRNA. The culture medium was entirely replaced 24 h following transfection, and the cells were subsequently maintained for an additional 48 h prior to further treatment. The cumulative duration from transfection to treatment was consistently documented as 72 h. Stable transfected cell lines were established through selection in a medium supplemented with 2 µg/mL puromycin (ST551, Beyotime) over a period of two weeks.

### 4.3 Wb

Total protein was extracted from cells utilizing a RIPA strong lysis buffer, which included 1 mm phenylmethylsulfonyl fluoride (PMSF, P0013, Beyotime) and PC201plus (Yeasen Biotechnology). The protein concentration was quantified using a BCA protein assay kit (P0011, Beyotime), and sample loading was standardized to ensure uniformity. Equivalent amounts of protein samples were denatured by boiling, subjected to separation via electrophoresis on 10% SDS‐PAGE gels (PG221, Epizyme), and subsequently transferred to PVDF membranes (IPVH00010, Millipore) employing the wet transfer method (250 mA, 90 min, 4°C). The membranes were blocked with 5% skimmed milk at room temperature for 1 h, followed by incubation with the appropriate primary antibodies (details provided in Table S2) overnight at 4°C. Following washes with TBST (PS103, Epizyme), the membranes were incubated with HRP‐conjugated secondary antibodies (rabbit/mouse, AS011/AS007, Abclonal) at room temperature for 1 h. After additional washes with TBST, signal development was performed using an ECL chemiluminescence kit (SQ202, Beyotime), and band intensity was quantified using ImageJ software.

### 4.4 RNA Extraction and qPCR

Total RNA was isolated utilizing the TRIzol reagent, followed by the synthesis of complementary DNA (cDNA) from the extracted RNA employing the PrimeScript RT Kit (TaKaRa, 9109) in accordance with the manufacturer's protocol. qPCR was conducted using the TB Green Premix Ex Taq II (TaKaRa, RR037A), with detection facilitated by a real‐time fluorescence quantitative PCR system. Glyceraldehyde 3‐phosphate dehydrogenase (GAPDH) served as the internal reference gene, and relative gene expression levels were quantified using the 2^(‐ΔΔCt) method. The sequences of the relevant primers are provided in Table S3.

### 4.5 IF

IF staining was conducted on cell slides or tissue sections. The samples were initially fixed using 4% paraformaldehyde at room temperature for 15 min, followed by three washes with phosphate‐buffered saline (PBS). Permeabilization was achieved through treatment with 0.1% Triton X‐100 for 10 min, succeeded by additional PBS washes. To prevent non‐specific antigen binding, a blocking step was performed with 5% bovine serum albumin (BSA) at room temperature for 30 min. Subsequently, primary antibodies, diluted as specified in Table S2, were applied and incubated overnight at 4°C in a humidified chamber. On the following day, the samples underwent three thorough PBS washes, each lasting 5 min. Species‐specific Alexa Fluor 488 or 594 conjugated secondary antibodies (AS053/AS037, Abclonal) were then administered and incubated at room temperature for 1 h, shielded from light, followed by an additional three PBS washes. Finally, cell nuclei were counterstained with DAPI staining solution (C1006, Beyotime) for 5 min at room temperature. After a final PBS rinse, the samples were mounted using an aqueous mounting medium. All samples were examined, and images were acquired utilizing a Zeiss LSM 900 confocal microscope. Subsequent image processing and fluorescence merging were conducted using ZEN software.

### 4.6 IHC

Paraffin‐embedded tissue sections were subjected to overnight incubation in a 60°C oven, followed by deparaffinization in two changes of xylene for 15 min each. The sections were subsequently rehydrated through a graded series of alcohols, with each step lasting 5 min, and were rinsed three times with PBS for 5 min per rinse. Antigen retrieval was conducted using a citrate sodium buffer (pH 6.0, 005000, Thermo Fisher) in a pressure cooker for 3 min. Following natural cooling to room temperature, the sections were washed again with PBS. To inhibit endogenous peroxidase activity, the sections were treated with a 3% hydrogen peroxide solution at room temperature for 25 min, shielded from light, followed by thorough washing with PBS. Appropriate primary antibodies (refer to Table S2 for detailed information) were applied to cover the tissue and incubated overnight in a humidified chamber at 4°C. After washing with PBS the following day, species‐specific horseradish peroxidase (HRP)‐labeled secondary antibodies were applied and incubated at room temperature for 1 h, followed by an additional PBS wash. Color development was conducted utilizing a DAB kit (ZSGB‐BIO), with the duration of development meticulously monitored under microscopic observation. The development process was terminated by rinsing with running water. Nuclei were counterstained with hematoxylin for a duration of 2 min, followed by differentiation with hydrochloric acid alcohol, and subsequently blued under running water. The sections underwent dehydration through a series of graded alcohols, were cleared in xylene, and ultimately mounted with neutral balsam. Each section was independently evaluated by two pathologists using a Nikon upright optical microscope. IHC scoring was carried out based on the intensity of staining and the proportion of positive cells.

### 4.7 Co‐IP

Cells were lysed using pre‐cooled IP lysis buffer, which included 1 mm PMSF, for 30 min at 4°C. This was followed by centrifugation at 12 000 rpm for 15 min at 4°C to obtain the supernatant. The supernatant underwent pre‐clearance by incubation with Protein A/G Agarose beads (Bio‐Rad) on a shaker at 4°C for 2 h to eliminate non‐specific binding. Following pre‐clearance, a portion of the supernatant was reserved as the Input group. The remaining sample was incubated with the target primary antibody (refer to Table S2 for antibody details) overnight at 4°C. Subsequently, an appropriate volume of Protein A/G Agarose beads was added, and the incubation was continued at 4°C for an additional 4 h to facilitate the formation of antigen‐antibody‐bead complexes. These complexes were washed four times with pre‐cooled IP lysis buffer, each wash lasting 5 min, to remove unbound proteins. Finally, 1× SDS loading buffer was added to the precipitate, which was then boiled in a water bath for 10 min to denature and dissociate the proteins. The supernatant was collected post‐centrifugation for Wb analysis.

### 4.8 Proteomics Sequencing

In this study, data‐independent acquisition (DIA) quantitative proteomics technology was utilized. Cellular proteins underwent lysis, reduction, alkylation, and purification through precipitation. Sequential digestion was conducted using trypsin enzymes. The resulting peptides were subjected to C18 desalting followed by pre‐fractionation via high‐performance liquid chromatography (HPLC). Subsequent analysis was carried out using an Orbitrap Astral mass spectrometer. Data processing was performed with DIA‐NN software, and protein quantitative values were normalized accordingly. Differentially expressed proteins were identified using an unpaired two‐tailed Student's *t*‐test (*p* < 0.05), and the findings were visualized through volcano plots, GO, and KEGG enrichment analysis. The quantitative proteomics sequencing experiment and a portion of the analytical work in this study were supported by Novogene Co., Ltd. (Beijing).

### 4.9 Measurement of Intracellular Copper Levels

The intracellular copper concentration was quantified using a Copper Assay Kit (Colorimetric, ab272528, Abcam). Briefly, approximately 1×10^7 treated NP cells were harvested and homogenized in 1 mL of distilled water. After centrifugation to remove debris, 50 µL of the supernatant was transferred to a flat‐bottom 96‐well plate. Then, 50 µL of Reagent A was added to each well, mixed thoroughly, and incubated at room temperature for 5 min. Subsequently, 50 µL of Reagent B was added, mixed, and incubated for another 5 min at room temperature. The absorbance was immediately measured at 570 nm using a microplate reader (Synergy 2, BioTek Instruments, USA). A standard curve was prepared concurrently using copper standard solutions provided in the kit. The intracellular copper concentration was calculated from the standard curve and normalized to the total protein concentration of the sample.

### 4.10 IDD Animal Model

Three‐month‐old male SD rats (weighing approximately 250–300 g), procured from Shanghai SLAC Laboratory Animal Co., Ltd., were randomly allocated into the following experimental groups, with six rats per group: Control, IDD, 10 µm Celecoxib, 20 µm Celecoxib, 30 µm Celecoxib, 40 µm Celecoxib, 50 µm Celecoxib, 50 µm Celecoxib + TTM, 50 µm Celecoxib + shHSP90, and 50 µm Celecoxib + shHSP90+ Elesclomol. The degeneration model was established by puncturing the Co6/7 IVD using a 21‐gauge needle, followed by the administration of 2 µL of the designated therapeutic agent. The animals were maintained in a specific pathogen‐free environment under a 12 h light/dark cycle at a temperature of 25°C, with unrestricted access to food and water. Radiographic and histological evaluations were conducted on samples collected at 4‐ and 8‐weeks post‐intervention. This study was conducted in accordance with ethical guidelines and received approval from the Ethics Committee of Tongji University (Approval No.: TJBB05022101).

### 4.11 Radiographic and Histological Assessment

Radiographic evaluations of the rat caudal vertebrae were conducted at 4‐ and 8‐weeks following surgery. Lateral X‐ray images were acquired using a small animal imaging system, while MRI scans were performed with a 3.0T MRI system (Philips). The DHI% was employed to quantitatively assess alterations in the intervertebral space, calculated as (D + E + F) × 100% / (A + B + C + D + E + F), where A, B, and C denote the anterior, middle, and posterior heights of the superior vertebral body, and D, E, and F correspond to the anterior, middle, and posterior heights of the intervertebral space, respectively [56]. IVD rehydration was evaluated using T2‐weighted imaging (3.0 T, GE Healthcare), and the MRI index was determined by multiplying the NP area by the average signal intensity [57]. For histological analysis, the harvested caudal spine segments were fixed in 4% paraformaldehyde (G1107, Servicebio) for 48 h, followed by decalcification in a 10% EDTA solution (10009617, China National Pharmaceutical Group Corp.) for 8 weeks. Subsequent processing involved rinsing under running water, dehydration through a graded ethanol series, clearing, and embedding in paraffin. Consecutive sections were stained with HE and SO to observe the morphology of the IVD and the distribution of proteoglycans, respectively. Finally, the IDD was assessed blindly and independently by two observers who were unaware of the group assignments, according to the established grading system [58].

### 4.12 Mitochondria‐Lysosome Co‐Localization Analysis

Cells were stained in accordance with the manufacturer's protocol utilizing MitoTracker Green (C1048, Beyotime) and LysoTracker Red (C1046, Beyotime), and subsequently, images were acquired through fluorescence microscopy.

### 4.13 CCK‐8 Assay

Cell cytotoxicity was assessed utilizing the CCK‐8 assay kit (C0037, Beyotime). Following centrifugation, logarithmically proliferating NP cells were plated in 96‐well plates at a density of approximately 1 × 10^4^ cells per well, with PBS added to the peripheral wells to mitigate edge effects. Upon cell adhesion, the supernatant was aspirated and replaced with a medium containing Celecoxib at varying concentrations (10, 20, 30, 40, 50, 75, and 100 µm). Each concentration was evaluated in a minimum of triplicate wells, and the experiments were independently replicated three times. After a 48 h incubation period, 10 µL of CCK‐8 solution was introduced into each well, and the plates were incubated for an additional hour in a humidified incubator set at 37°C with 5% CO_2_. Subsequently, the absorbance at 450 nm was immediately measured using a microplate reader.

### 4.14 Lipid Peroxidation

The lipid peroxidation product MDA in NP cells was measured using a commercial MDA Assay Kit (ab118970, Abcam) following the manufacturer's instructions. Briefly, NP cells were homogenized in MDA Lysis Buffer supplemented with BHT. The homogenate was centrifuged at 13 000 × g for 10 min at 4°C to collect the supernatant. The sample supernatant was mixed with TBA reagent. The mixture was incubated at 95°C for 60 min in a dry bath and then cooled on ice for 10 min. The absorbance of the resulting MDA‐TBA adduct was measured at 532 nm using a microplate reader. The MDA concentration in each sample was determined by interpolation from a standard curve.

### 4.15 Detection of GSH/GSSG Ratio

In accordance with the manufacturer's protocol, intracellular concentrations of total GSH and GSSG were quantified utilizing a GSH assay kit (G263, Dojindo). Relative concentrations were subsequently analyzed using a microplate reader (BioRad). The ratio of GSH to GSSG was then determined.

### 4.16 Enzyme‐Linked Immunosorbent Assay (ELISA)

The concentrations of IL‐6 and TNF‐α in IVD tissue were quantified utilizing ELISA systems. The ELISA kits for IL‐6 (KET9007) and TNF‐α (KET9007) were procured from Abbkine, Wuhan, China.

### 4.17 Statistical Analysis

All statistical analyses and graphical representations were conducted using GraphPad Prism software (version 9.5.0). Measurement data are expressed as the mean ± standard deviation (SD). Data for each group were obtained from a minimum of three independent replicate experiments, with specific sample sizes (*n*‐values) provided in the figure legends. Prior to data analysis, the normality of the data was assessed using the Shapiro–Wilk test, and homogeneity of variance was evaluated using the Brown‐Forsythe test. For datasets adhering to a normal distribution with equal variances, comparisons between two groups were conducted using unpaired *t*‐tests, while comparisons among multiple groups were performed using one‐way analysis of variance (one‐way ANOVA). In cases where significant differences were detected, Tukey's post hoc test was employed for multiple comparisons. Statistical significance was determined at a threshold of *p* < 0.05, with significance levels denoted in the figures as ^*^ *p* < 0.05, ^**^ *p* < 0.01, ^***^ *p* < 0.001; “ns” denotes no significant difference.

## Author Contributions

Y.G., H.X., S.B., Y.Z., and N.S. contributed to conceptualization; Y.G., H.X., S.B., and Y.Z. to methodology; Y.G., H.X., S.B., B.W., Y.H., and B.Y. to fabrication and investigation; Y.G., H.X., S.B., N.S., and D.W. to supervision and writing – original draft; and H.Z., Z.S.C., N.S., and Z.B. to writing – review and editing. Y.G., H.X., S.B., Y.Z., and N.S. are co‐first authors, while H.Z., N.S., Z.S.C., and Z.B. are co‐corresponding authors.

## Funding

This work was funded by the program of Science and Technology Innovation Action of Science and Technology Commission of Shanghai Municipality (STCSM) (No. 24SF1903301), The Medical Discipline Construction Program of Shanghai Pudong New Area Health Commission (the Key Disciplines Program) (PWZxk2022‐21), and Science and Technology Development Fund of Shanghai Pudong New Area (PKJ2024‐Y22).

## Ethics Statement

The study was reviewed and approved by the Ethics Committee of East Hospital Affiliated to Tongji University (DF2025(CR)‐01 and TJBB05022101).

## Consent

All authors gave their consent for publication.

## Conflicts of Interest

The authors declare no conflicts of interest.

## Supporting information




**Supporting File**: advs75527‐sup‐0001‐SuppMat.pdf.

## Data Availability

The data that support the findings of this study are available from the corresponding author upon reasonable request.
