# Supplementary material for: Drug‐Induced Cuproptosis Defines the Therapeutic Window of Celecoxib in Intervertebral Disc Degeneration via the HSP90‐RBX1 Axis
Source: Adv Sci (Weinh). 2026 May 4;13(42):e75527. doi: 10.1002/advs.75527 (PMC13335710; doi:10.1002/advs.75527)
Supplement: Supplementary file 1 — Supporting File: advs75527‐sup‐0001‐SuppMat.pdf. [file ADVS-13-e75527-s001.pdf]

Supporting Information

Drug-Induced Cuproptosis Defines the Therapeutic Window of Celecoxib in Intervertebral Disc Degeneration via the HSP90-RBX1 Axis

Youfeng Guo, Hongju Xiao, Shenghao Ba, Yu Zhou, Bijun Wang, Bin Yu, Yufeng Huang, Haihong Zhao, Zhefan Stephen Chen, Na Shen, Zhaoyu Ba, Desheng Wu

Table S1. Clinical information of human degenerative disc samples from 6 patients.

	Patient 1	Patient 2	Patient 3	Patient 4	Patient 5	Patient 6
Sex	Female	Male	Male	Female	Male	Female
Age	35	30	36	65	70	68
Diagnosis	Fracture	Fracture	Fracture	LSS	LSS	LSS
Level	L4/5	L5/S1	L5/S1	L4/5	L5/S1	L4/5
Grades	II	II	II	IV	IV	IV

LSS, Lumbar Spinal Stenosis

Table S2. Antibodies used in this study

Antibody	Manufacturer	Catalog No.
COL2	Proteintech	28459-1-AP
ADAMTS4	Proteintech	11865-1-AP
ubiquitin	Proteintech	80992-1-RR
β-actin	Abclonal	AC026
Flag	Cell Signaling Technology	14793
Myc	Cell Signaling Technology	2276
HA	Cell Signaling Technology	3724
GST	Cell Signaling Technology	2622
HSP90	Santa Cruz Biotechnology	SC-13119
RBX1	Abclonal	A14500
COMMD1	Proteintech	11938-1-AP
ATP7B	Proteintech	19786-1-AP
USP15	Proteintech	14354-1-AP
ACAN	Proteintech	13880-1-AP

COL2, Collagen type II; ADAMTS4, A disintegrin and metalloproteinase with thrombospondin motifs 4; HSP90, Heat shock protein 90; RBX1, RING-Box 1; COMMD1, Copper metabolism MURR1 domain 1; ATP7B, Adenosine triphosphatase copper transporting beta; USP15, Ubiquitin specific peptidase 15; ACAN, Aggrecan.

Table S3. The PCR primers used in this study.

	primer sequences (forward)	primer sequences (reverse)
HSP90	5′-CCCAACAAGCAAGACCGAACC-3′	5′-CCAGCCTGCAAAGCCTCCATG-3′
RBX1	5′-TTGGGGAGTCTGTAACCACG-3′	5′-CTCTGTTGTCCAAGGGGCAC-3′
COMMD1	5′-CAGTCACGGCACTCAACTCA-3′	5′-TCAGCCCGCTGATACTCTCT-3′
ATP7B	5′-CCCAGAACGGCGTCCTAA-3′	5′-CGCACAGCACACCATCAA-3′
ADAMTS4	5′-CGCTGAGTAGATTCGTGGAGAC-3′	5′-AGTTGACAGGGTTTCGGATGC-3′
COL2	5′-TCCTAAGGGTGCCAATGGTGA-3′	5′-AGGACCAACTTTGCCTTGAGGAC-3′
β-actin	5′-TGAACGGGAAGCTCACTGG-3′	5′-TCCACCACCCTGTTGCTGTA-3′

COL2, Collagen type II; ADAMTS4, A disintegrin and metalloproteinase with thrombospondin motifs 4; HSP90, Heat shock protein 90; RBX1, RING-Box 1; COMMD1, Copper metabolism MURR1 domain 1; ATP7B, Adenosine triphosphatase copper transporting beta.

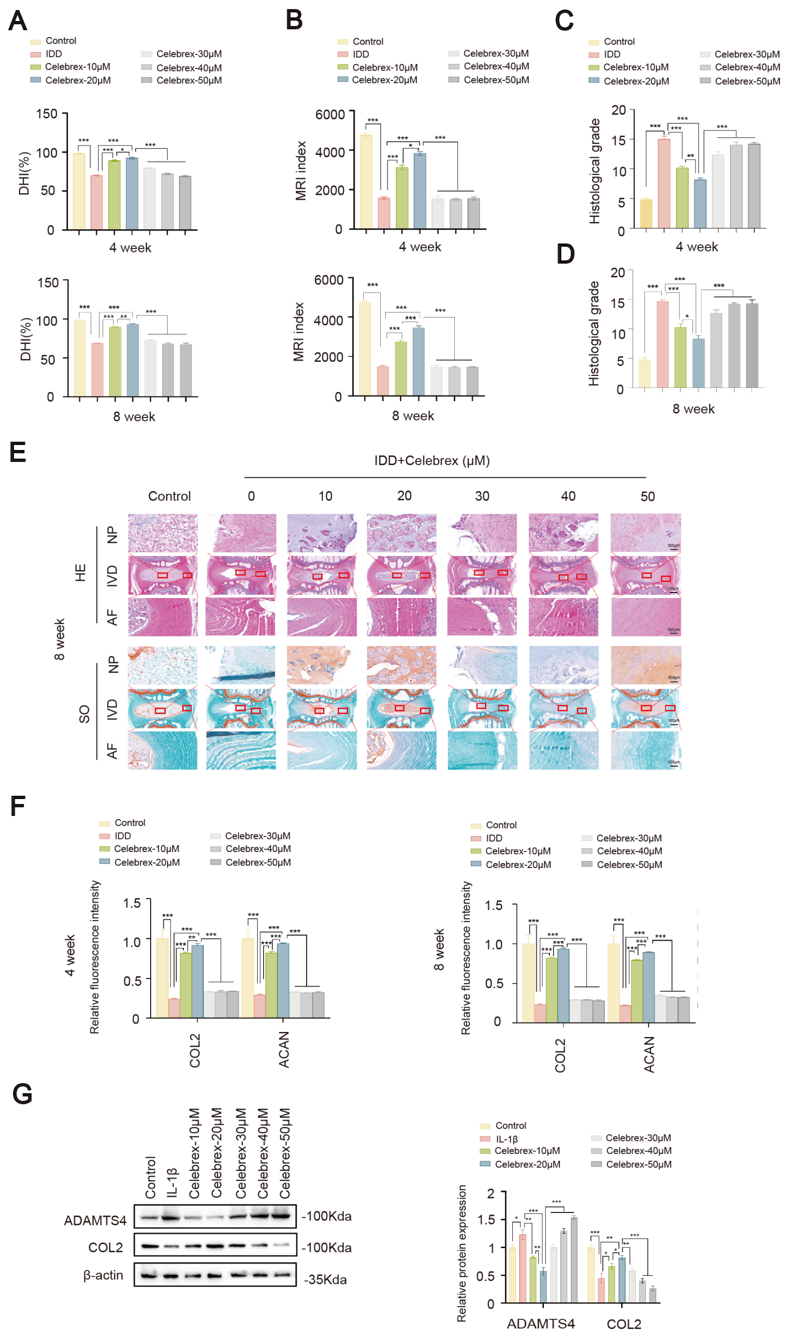

Fig. S1 Effects of different concentration gradients of celecoxib on IDD. A: Quantitative analysis of DHI index in IVDs at 4- and 8-weeks post-intervention (n = 6). B: Quantitative analysis of MRI index in IVDs at 4- and 8-weeks post-intervention (n = 6). C-D: Quantitative histological scores of IVDs at 4- and 8-weeks post-intervention (n = 6). E: Representative HE staining and SO staining images at 8 weeks post-intervention (n = 6). F: Quantitative analysis of COL2 and ACAN IF staining in IVDs at 4- and 8-weeks post-intervention (n = 6). G: Protein levels of ADAMTS4 and COL2 in IVD tissues detected by Wb at 4- and 8-weeks post-intervention, with semi-quantitative analysis of band grayscale values using ImageJ (n = 3). All data are presented as mean ± SD. Comparisons between two groups were performed using an unpaired two-tailed Student's t-test or ANOVA followed by Tukey's post hoc test. A P-value less than 0.05 was considered statistically significant. * indicates $p < 0.05$, ** indicates $p < 0.01$, *** indicates $p < 0.001$, while "ns" indicates a lack of statistical significance. IDD, Intervertebral disc degeneration; DHI, Disc Height Index; IVD, Intervertebral Disc; MRI, Magnetic Resonance Imaging; HE, Hematoxylin and Eosin; SO, Safranin O; COL2, Type II Collagen; ACAN, Aggrecan; IF, Immunofluorescence; ADAMTS4, A Disintegrin and Metalloproteinase with Thrombospondin Motifs 4; SD, Standard Deviation; Wb, Western blot; ANOVA, one-way analysis of variance.

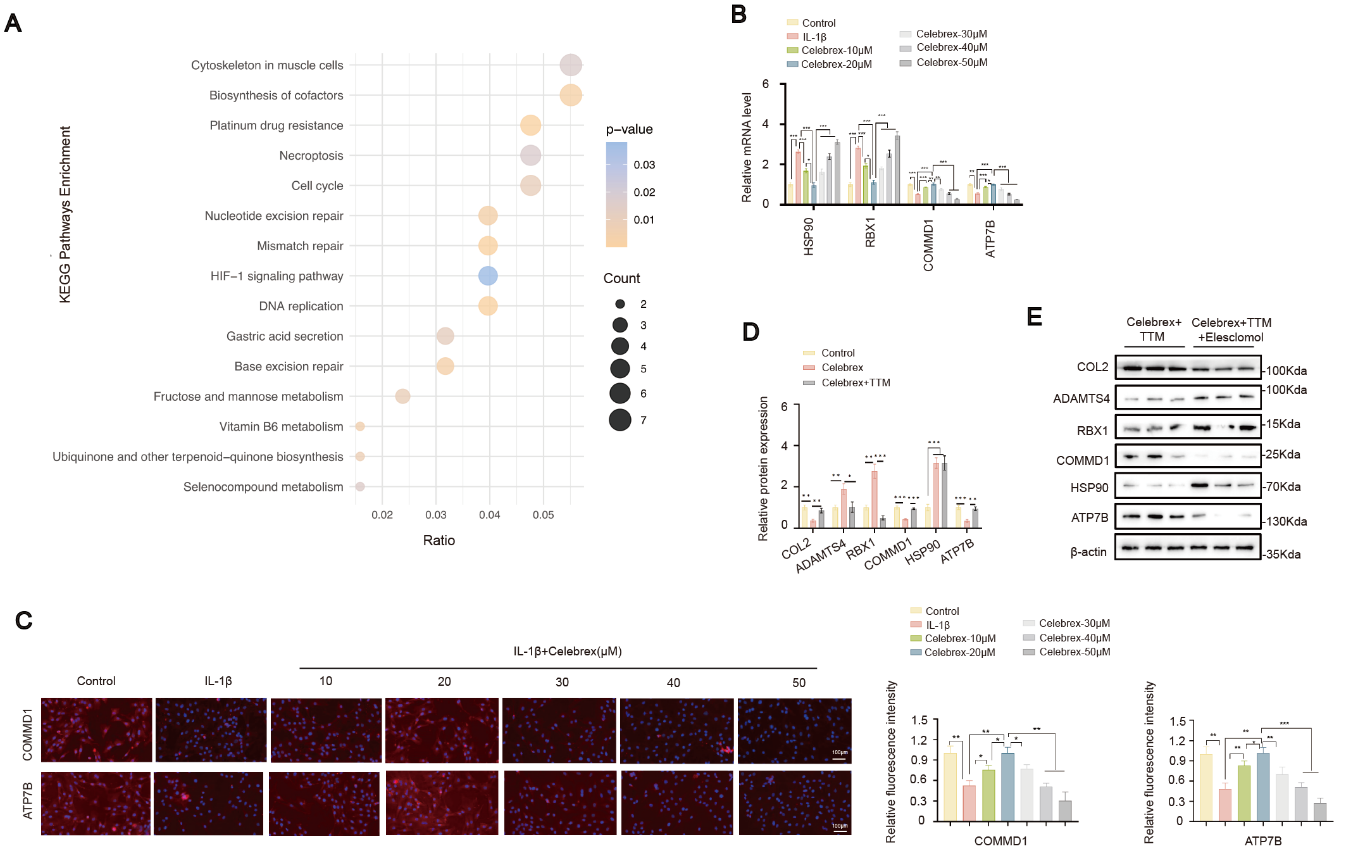

Fig. S2 Proteomic analysis and validation of the mechanism underlying the attenuated therapeutic effect of high-concentration celecoxib. A: Proteomics sequencing comparison between NP cells treated with high-concentration celecoxib (50µM) and low-concentration celecoxib (20µM), followed by KEGG analysis of differentially expressed proteins. B: mRNA levels of ATP7B, HSP90, COMMD1, and RBX1 detected after treatment with different concentrations of celecoxib for 48h in the presence of IL-1β (10 ng/ml). C: Protein levels of COMMD1 and ATP7B analyzed by IF staining after treatment with different concentrations of celecoxib for 48h in the presence of IL-1β (10 ng/ml), with semi-quantitative analysis using ImageJ (n = 3). D: Protein levels of ATP7B, HSP90, COMMD1, RBX1, COL2, and ADAMTS4 detected after treatment with or without TTM for 48h in the presence of celecoxib (50 µM), with semi-quantitative analysis of band grayscale values using ImageJ (n = 3). E: We treated NP cells with two interventions: "50 µM Celecoxib + TTM" and "50 µM Celecoxib + TTM + Elesclomol." Key phenotypic indicators of degeneration and cuproptosis were measured by Wb, with semi-quantitative analysis using ImageJ (n = 3). All data are presented as mean ± SD. Comparisons between two groups were performed using an unpaired two-tailed Student's t-test or ANOVA followed by Tukey's post hoc test. A P-value less than 0.05 was considered statistically significant. * indicates $p < 0.05$, ** indicates $p < 0.01$, *** indicates $p < 0.001$, while "ns" indicates a lack of statistical significance. NP, Nucleus Pulposus; KEGG, Kyoto Encyclopedia of Genes and Genomes; ATP7B, ATPase Copper Transporting Beta; HSP90, Heat Shock Protein 90; COMMD1, Copper Metabolism MURR1 Domain-containing protein 1; RBX1, RING Box Protein 1; IL-1β, Interleukin-1 beta; IF, Immunofluorescence; COL2, Type II Collagen; ADAMTS4, A Disintegrin and Metalloproteinase with Thrombospondin Motifs 4; TTM, Tetrathiomolybdate; SD, Standard Deviation; ANOVA, one-way analysis of variance.

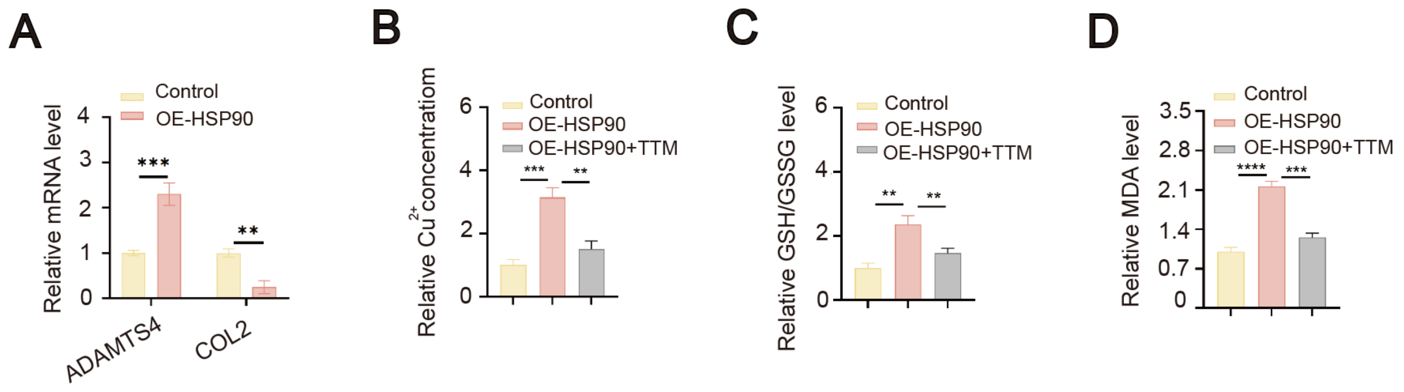

Fig. S3 HSP90 drives NP cell degeneration via cuproptosis. A: In vitro analysis of related (ADAMTS4 and COL2) levels by qPCR in NP cells transfected as indicated. B-D: The cuproptosis levels in NP cells transfected as indicated were determined by Cu^{2+} (B), GSH/GSSG (C) and MDA (D) levels. All data are presented as mean ± SD. Comparisons between two groups were performed using an unpaired two-tailed Student's t-test or ANOVA followed by Tukey's post hoc test. A P-value less than 0.05 was considered statistically significant. * indicates $p < 0.05$, ** indicates $p < 0.01$, *** indicates $p < 0.001$, while "ns" indicates a lack of statistical significance. HSP90, Heat Shock Protein 90; GSH, Reduced Glutathione; GSSG, Oxidized Glutathione; MDA, Malondialdehyde; qPCR, Quantitative Polymerase Chain Reaction; ADAMTS4, A Disintegrin and Metalloproteinase with Thrombospondin Motifs 4; COL2, Type II Collagen; TTM, Tetrathiomolybdate; ANOVA, one-way analysis of variance; SD, Standard Deviation.

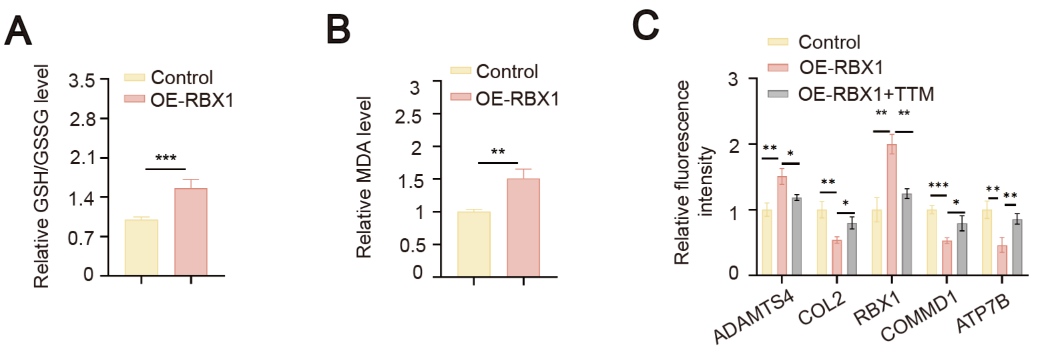

Fig. S4 RBX1 drives NP cell degeneration via cuproptosis. A-B: The cuproptosis levels in NP cells transfected as indicated were determined by GSH/GSSG (A) and MDA (B) levels. C: In vitro analysis of related (ADAMTS4 and COL2), cuproptosis (ATP7B and COMMD1), HSP90 and RBX1 levels were determined by cellular IF staining of RBX1 overexpression with or without TTM-treated NP cells. All data are presented as mean ± SD. Comparisons between two groups were performed using an unpaired two-tailed Student's t-test or ANOVA followed by Tukey's post hoc test. A P-value less than 0.05 was considered statistically significant. * indicates $p < 0.05$, ** indicates $p < 0.01$, *** indicates $p < 0.001$, while "ns" indicates a lack of statistical significance. ATP7B, ATPase Copper Transporting Beta; COMMD1, Copper Metabolism MURR1 Domaincontaining protein 1; RBX1, RING Box Protein 1; GSH, Reduced Glutathione; GSSG, Oxidized Glutathione; MDA, Malondialdehyde; ADAMTS4, A Disintegrin and Metalloproteinase with Thrombospondin Motifs 4; COL2, Type II Collagen; TTM, Tetrathiomolybdate; ANOVA, one-way analysis of variance; SD, Standard Deviation.

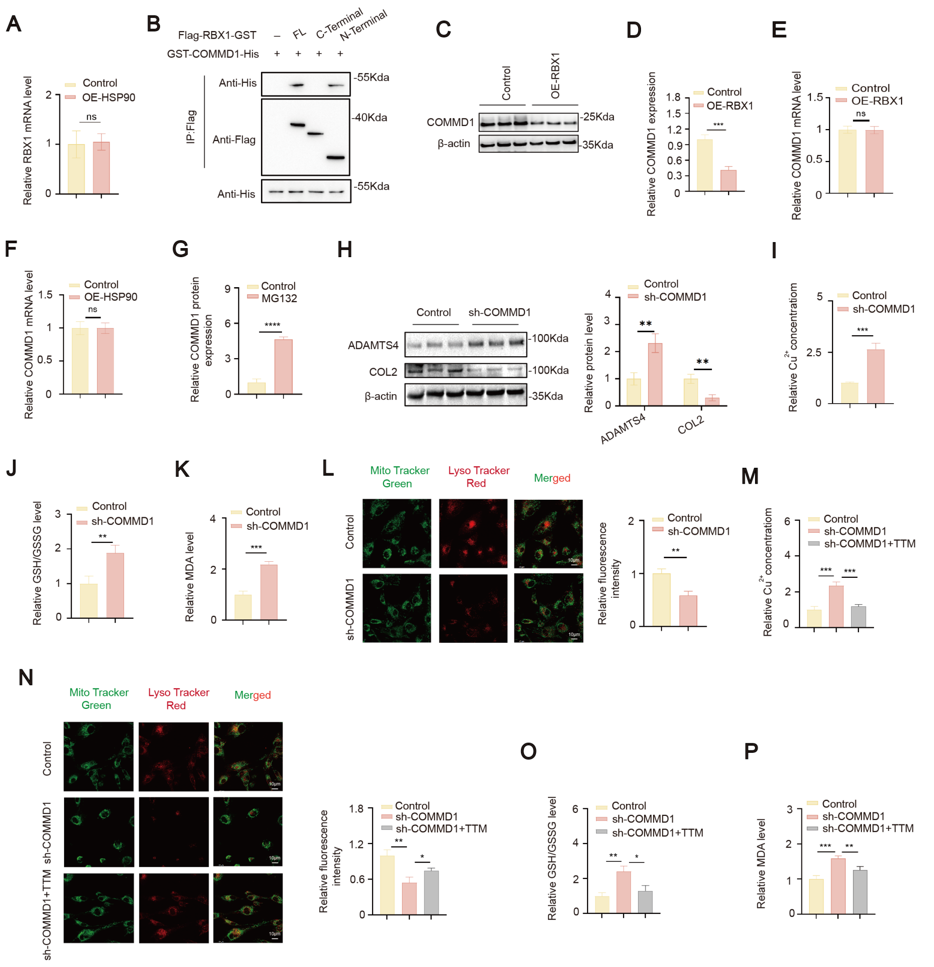

Fig. S5 Functional regulation of the HSP90-RBX1-COMMD1 axis. A: In vitro analysis of RBX1 levels were determined by cellular qPCR of HSP90-overexpression NP cells. B: NP cells were co-transfected with HisCOMMD1 WT and Flag-RBX1 WT or domain overexpression plasmid. Cell lysates were subjected to IP, followed by Wb with antibodies against His and Flag. C-E: In vitro analysis of COMMD1 levels were determined by cellular Wb (C-D) and qPCR (E) of RBX1-overexpression NP cells. F: In vitro analysis of COMMD1 levels were determined by qPCR of HSP90-overexpression NP cells. G: Semi-quantitative analysis of COMMD1 expression in NP cells treated with MG132. H: In vitro analysis of related (ADAMTS4 and COL2) levels was determined by cellular Wb of control and sh-COMMD1 NP cells. I-P: The cuproptosis levels in NP cells transfected as indicated were determined by Cu^{2+} (I and M), mitophagy (L and N), GSH/GSSG (J and O), and MDA (K and P) levels. All data are presented as mean ± SD. Comparisons between two groups were performed using an unpaired two-tailed Student's t-test or ANOVA followed by Tukey's post hoc test. A P-value less than 0.05 was considered statistically significant. * indicates $p < 0.05$, ** indicates $p < 0.01$, *** indicates $p < 0.001$, while "ns" indicates a lack of statistical significance. ATP7B, ATPase Copper Transporting Beta; COMMD1, Copper Metabolism MURR1 Domain-containing protein 1; RBX1, RING Box Protein 1; GSH, Reduced Glutathione; GSSG, Oxidized Glutathione; MDA, Malondialdehyde; qPCR, Quantitative Polymerase Chain Reaction; HSP90, Heat Shock Protein 90; NP, Nucleus Pulposus; Wb, Western blot; TTM, Tetrathiomolybdate; ANOVA, one-way analysis of variance; SD, Standard Deviation.

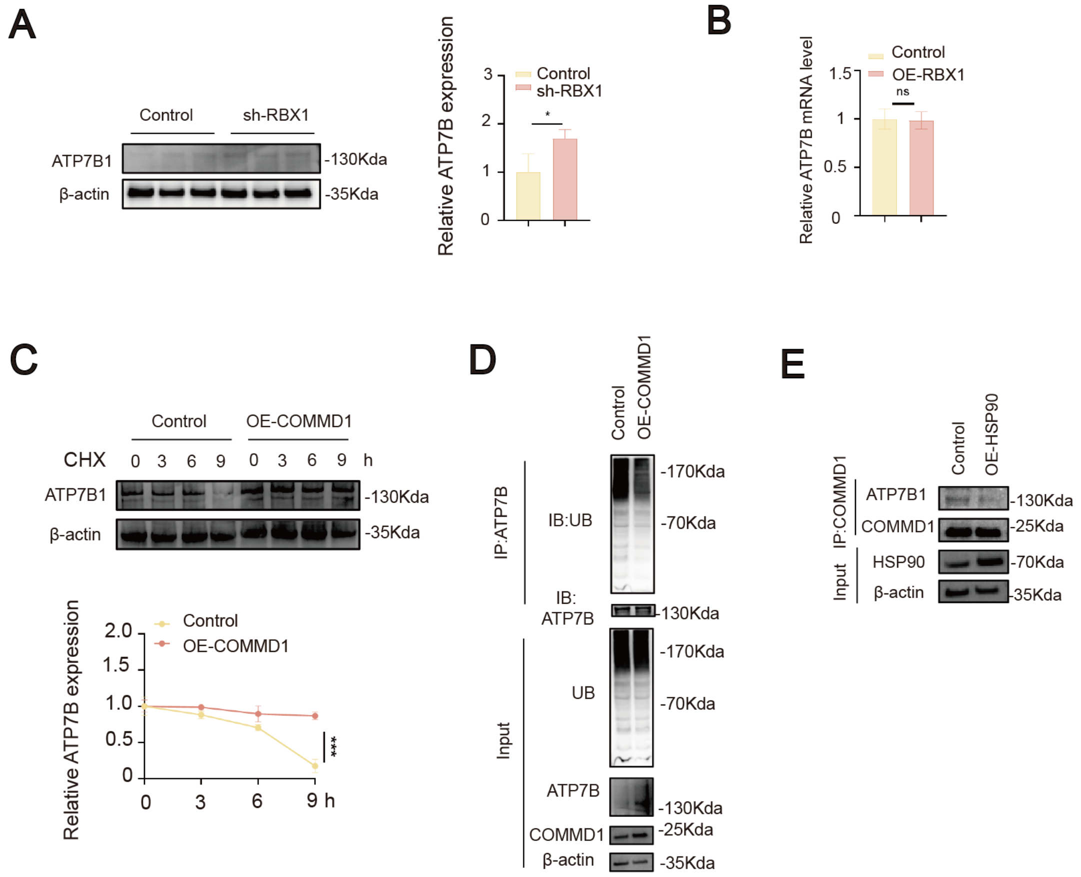

Fig. S6 Functional regulation of the RBX1-COMMD1-RBX1 axis. A-B: In vitro analysis of ATP7B levels were determined by cellular Wb (A) and qPCR (B) of NP cells. C: NP cells were transfected with Control or COMMD1-overexpressing plasmid, and then treated with CHX (50 μg/mL) and collected at the indicated times. Cell lysates were subjected to Wb with antibodies against ATP7B. D: COMMD1-control and overexpression NP cells were treated with MG132 (20 μM for 8 h) before collecting. Cell lysates were subjected to d-IP. E: Cell lysates of control and HSP90-overexpression NP cells were immunoprecipitated with COMMD1 antibodies, and IB assays were performed using ATP7B, COMMD1 or HSP90 antibodies. All data are presented as mean ± SD. Comparisons between two groups were performed using an unpaired two-tailed Student's t-test or ANOVA followed by Tukey's post hoc test. A P-value less than 0.05 was considered statistically significant. * indicates $p < 0.05$, ** indicates $p < 0.01$, *** indicates $p < 0.001$, while "ns" indicates a lack of statistical significance. ATP7B, ATPase Copper Transporting Beta; COMMD1, Copper Metabolism MURR1 Domain-containing protein 1; RBX1, RING Box Protein 1; qPCR, Quantitative Polymerase Chain Reaction; HSP90, Heat Shock Protein 90; NP, Nucleus Pulposus; Wb, Western blot; ANOVA, one-way analysis of variance; SD, Standard Deviation.
